# Designing for dissemination through community advisory board engagement in an implementation mapping process: A case study

**DOI:** 10.1017/cts.2025.10065

**Published:** 2025-06-09

**Authors:** Gabriella M. McLoughlin, Yerusalem Yohannes, Molly Kerstetter, Omar Martinez, Alex Dopp, Jennifer O. Fisher, Maura Kepper, Rachel G. Tabak, Ross C. Brownson

**Affiliations:** 1 Temple University College of Public Health, Philadelphia, PA, USA; 2 College of Medicine, University of Central Florida, Orlando, FL, USA; 3 RAND, Santa Monica, CA, USA; 4 Prevention Research Center, Brown School at Washington University in St. Louis, St. Louis, MO, USA; 5 Division of Public Health Sciences and the Alvin J. Siteman Cancer Center, Washington University School of Medicine, St. Louis, MO, USA

**Keywords:** Implementation science, community engagement, designing for dissemination, sustainability, equity

## Abstract

**Introduction:**

The purpose of this study was to document the development of a Community Advisory Board (CAB) to enhance equitable dissemination of research findings within an implementation mapping study to enhance equitable impact of Universal School Meals (USM) through the Designing for Dissemination and Sustainability (D4DS) process.

**Methods:**

The D4DS process comprises 7 key elements to facilitate meaningful dissemination. To accomplish Step 1: Identify Partners, the research team conducted snowball recruitment methods within the local Philadelphia community and with existing connections. To Empathize and Outline the Problem (Step 2) and Understand the Context (Step 3), an interest meeting was held followed by monthly meetings. Our team Confirmed and Co-designed the Product (Step 4) and Developed the Dissemination Plan (Step 5) through collaborative brainstorming sessions. Finally, we started the Iterative Evaluation (Step 6) and Plan for Sustainability (Step 7) by administering a baseline and follow-up survey measuring CAB members’ perceived utility, effectiveness, and sustainability of the board.

**Results:**

The final CAB included 8 members. The co-created dissemination products and plan comprised a 2-page infographic, social media toolkits, and a webinar slide deck, which were disseminated locally by the research team via presentations, websites, and email communication, in spring 2024. Initial findings from baseline and follow-up surveys indicated that CAB members benefited from skill development, compensation, writing credit, and autonomy in dissemination designing.

**Conclusions:**

Sharing power and decision-making enhanced the capacity for local-level dissemination, which is much needed to advance the science of community partnerships.

## Introduction

One of the primary challenges to meaningful translation research is a lack of engagement with community members and populations affected by public health interventions, leading to limited buy-in from those who deliver and receive public health interventions[1,2]. An effective approach to building partnerships with community members is through the establishment of a Community Advisory Board (CAB)[3–6]. A CAB serves as a dynamic team of diverse, relevant representatives of a specific community of interest. It creates a collaborative space where individuals from various organizations and affiliations come together with a shared purpose in mind. Although CABs can support a wide range of objectives, their overarching goal remains consistent: to tap into the valuable expertise of relevant individuals within a community. This collaborative approach ensures that the community’s unique insights and perspectives are at the forefront of the decision-making process[6,7].

Prior research highlights several key best practices such as co-creating expectations for both the research team and CAB members, establishing meeting times and locations that are accessible for all members, providing compensation appropriate for the time commitment and contributions, sharing data and study progress, and prioritizing opportunities for collaborative dissemination of findings[3,5,6,8–10]. Although the currently available academic literature provides best practices and examples of long-term collaborations to successfully implement interventions[3,4,11,12], little is documented about how best to utilize CAB member strengths and lived experiences in dissemination of research evidence. This is a gap in dissemination and implementation (D&I) science, as a clearer understanding of how to design for dissemination with CAB members can enhance the impact of research and improve sustainability of community engagement.

To address gaps in translation of research findings within public health, the Designing for Dissemination and Sustainability (D4DS) process was developed as an evidence-informed framework to help researchers “plan for the end in mind”[13]. D4DS helps balance research and practice considerations by acknowledging that active dissemination to relevant audiences should be the responsibility of the research team in collaboration with research partners. Careful attention to dissemination is especially needed given that peer-reviewed articles are often inaccessible or difficult to interpret or apply for those outside academia[14]. Most importantly, by moving beyond traditional dissemination methods, the D4DS process helps teams to consider possible barriers to and opportunities for sustainable and equitable dissemination to vulnerable populations.

To advance the use of D4DS among researchers and practitioners, its developers created an online webtool planner that allows collaboration with team members to co-design and evaluate a dissemination plan[15]. This process comprises 7 primary steps: Identify partners; Empathize and Outline the Problem; Understand the Context; Confirm and Co-design Your Product; Develop Dissemination Plan; Plan for Sustainability; and Evaluate Iteratively. The overarching goal of the D4DS process is to arrive at a clear dissemination plan to deliver research evidence beyond traditional academic channels, which can be evaluated for its impact over time. However, few published examples of D4DS are available and, to our knowledge, none that apply D4DS within a community-based implementation science project. Therefore, the primary research questions guiding this study were 1) How can researchers meaningfully build a CAB to enhance implementation science research? and 2) How can the D4DS process be utilized in collaboration with a CAB to enhance equitable dissemination of research evidence?

## Materials and methods

### Context for the study

Food insecurity remains a critical public health concern in Philadelphia, across the United States, and globally. It also disproportionately impacts low-income populations and those who identify as a minoritized racial or ethnic group, further marginalizing vulnerable groups. Universal School Meals (USM) provides greater access to free nutritious meals for students in schools serving high-poverty populations[16–19]. This provision has demonstrated success in reducing food insecurity, improving dietary intake, and reducing risk for childhood overweight and obesity[18,20]. Unfortunately, USM implementation varies across different settings and is often more difficult among schools serving socially and economically marginalized populations[21–23]. Challenges include difficulty recruiting and retaining enough staff to operate these programs (given low district budgets to offer competitive pay), lack of funding to build full-service kitchens in older buildings (given limited district budget for building renovations)[24,25], stigma faced among low-income students and families related to participation in federal programs[26], and less access to affordable healthy food within the neighborhood with families opting for less healthy options that are cheaper[27], thus competing with the mission of USM to provide nutritious meals. Accordingly, applying innovative, community-engaged methods to improve implementation of policies such as USM is critical[28].

### Setting

This case study took place over the 2023–2024 academic year within the city of Philadelphia, United States. Our research team sought to develop a CAB to collaborate with our research team throughout a 5-year, National Institutes of Health-funded study that uses community-engaged implementation mapping (K01 HL166957, principal investigator [PI] GMM) in collaboration with the School District of Philadelphia (SDP) to improve the implementation and equitable impact of USM (Temple University IRB# 28959). We began meeting monthly with the school district in fall 2022 to plan the recruitment and data collection process; staff from the Division of Food Services, the Office of Research and Evaluation, and the city’s Supplemental Nutrition Assistance Program Education (SNAP-ED) office attend regularly. This study aligned with the first year of the 5-year project to ensure CAB involvement throughout each aspect of the study. The PI’s college did not have an office of community engagement at the time of this study, thus the PI and research team sought to develop a CAB from scratch. This warranted additional effort and leadership from the research team to engage community partners, which we hope demonstrated our commitment to their involvement.

Implementation mapping is a collaborative process whereby researchers and practitioners work together to co-create an implementation strategy with the goal of improving implementation of evidence-based interventions and practices[29,30]. The overarching goal of this implementation mapping process is to improve the implementation and equitable impact of the USM provision across the SDP in collaboration with community partners (i.e., school teachers and staff, students, parents, school district representatives, CAB members). This process comprises 5 key steps: 1) Conduct a needs assessment, 2) determine implementation and performance outcomes, 3) develop implementation strategies, 4) develop protocols for implementation, and 5) evaluate strategies[29]. Collectively, this process can achieve meaningful change in complex systems such as SDP[29,31]. Formal research activity for the community-engaged implementation mapping study began in Fall 2023 with a district-wide needs assessment, the findings of which are reported elsewhere[32]. This step entailed working with 8 schools across the district (6 elementary-middle; 2 high), observing school mealtimes, and conducting interviews and survey data collection with students, parents, food service staff, teachers, administrators, and other staff at these schools. This provided a comprehensive view into the implementation of USM grounded in the voices of its implementers and recipients.

As planned during the conceptualization of the overarching study, we sought to recruit and retain a CAB to support us in enhancing the data collection, analysis, and dissemination procedures of the entire project. A key focus of this engagement in the first year was to strengthen dissemination beyond peer-reviewed articles; we wanted to draw upon community members’ lived experiences and expertise to guide dissemination locally which would hold us as researchers accountable to the community engagement process. Given the current lack of infrastructure for community engagement within the PI’s institution, we felt it appropriate to have realistic expectations for CAB involvement. According to the Centers for Disease control principles of community engagement[33], we prepared for the CAB to initially serve in a more consultant role with the intention of increasing engagement in future years of the project. The D4DS process occurred in parallel with the first step of the implementation mapping study to support ongoing dissemination of research findings. In accordance with the D4DS process, our methods and practices aligned specifically with each of the seven steps in the model, which are briefly outlined in Table 1 and described later.

**Table 1.** Designing for dissemination and sustainability (D4DS) action items and associated data source (D)/Practice (P)

Action Item	Data Source (D)/Practice (P)^a^	Time Frame
Identify partners	Review of existing CAB toolkits **(P)**Convenience sample of board members; snowball sample **(P)**Compensation for CAB members **(P)**	August-October 2023One-time stipend for full year in April 2024
Empathize and Outline the Problem	Informational meeting to interested individuals **(P)**Data/Research shared in meetings during Fall 2023 **(P)**	September 2023December 2023; January, February 2024
Understand the Context	Review of existing literature on food insecurity and disparities in Philadelphia **(P)**Spring 2024 Meetings: identifying contextual considerations for dissemination (i.e., populations of interest, channels of dissemination); sharing study updates **(P)**	June-September 2023February-April 2024
Confirm and Co-design Your Product	February 2024 meeting: planning for dissemination **(P)**Completing action plan worksheets **(D)**	February 2024February-March 2024
Develop Dissemination Plan	Working plan documents; online collaboration **(D)**Final products co-developed during and outside meetings **(P)**	January-May 2024May 2024
Plan For Sustainability	Baseline and follow-up survey of CAB member perceived impact **(D)**Monthly CAB meeting notes to guide future efforts **(D)**	November 2023; March 2024Monthly in 2023-2024
Evaluate Iteratively	Analysis of survey findings; co-creating plan for Year 2 based on reflection of earlier steps **(D)**	April-May 2024

**Note:** ^a^Data signifies new data collected through the CAB evaluation plan; Practice refers to steps taken by the research team to build capacity through the D4DS process.

### Identify partners

Before recruiting members, we sought guidance from CAB toolkits established by reputable sources including the National Resource Center for Refugees, Immigrants, and Migrants[11]; The Southern California Clinical and Translational Science Institute[34]; the Patient-Centered Outcomes Research Institute (PCORI),[7], and the Urban Institute[35]. These toolkits provided initial guidelines for developing and sustaining a CAB. The toolkits also emphasized understanding potential needs for member participation so we could address these by providing compensation for their time; limiting the group to 8–10 members to encourage discussions in an intimate environment; rotating meetings between virtual and in-person with an agreed upon location; and providing benefits for their membership such as skill building, writing credit, and networking opportunities[7,34].

Recruitment followed three key steps. First, we developed a flyer using graphic design software Canva® and an interest form on Google Forms, which we emailed to local networks and organizations within Philadelphia such as the Philadelphia Higher Education and Neighborhood Network Development (PHENND), Decolonize Philly, the Philadelphia Food Educators Network (part of the University of Pennsylvania), and networks that representatives from these organizations to recruit individuals who may be interested in joining the CAB. We sought the input from individuals working to address food insecurity and advocacy thus these organizations were an ideal fit for recruitment. Second, we conducted snowball sampling from these larger organizations and our ongoing study, and we ultimately identified an organization run by students who could represent students within the population. Our implementation mapping data collection process also directed us to a parent representative who participated in the study and had an interest in continuing the process of the study’s development. A total of 29 individuals completed the form expressing interest in either participating in the CAB or learning more ways to stay involved throughout the process. Third, we held an informational meeting in September 2023 to inform interested participants on the background of the study and the expectations of their participation. Of the 29 individuals, we asked those who still wished to be engaged to email the research team after the meeting. Those who confirmed interest through a consent form were invited to attend the first meeting in October 2023. The final composition of members is reported in the results section. Based on feedback from the PI’s mentoring team and guidance from the field[7], we provided CAB members with compensation of $50 for every meeting attended in the form of an e-visa gift card as compensation for their time and contributions (totaling $300 in the first year) in addition to meals during CAB meetings.

### Empathize and outline the problem

These initial steps were grounded in guidance from the national-level organizations for partnered research[7,35,36]. The informational meeting provided CAB members with a comprehensive overview of our collaboration with SDP and the goals of the first year, which comprised a mixed methods needs assessment of USM. We presented our study background, followed by CAB goals, expectations, project timeline, and compensation/benefits of CAB participation. This helped inform interested members about our values and intentions regarding building the advisory board.

Once we had the members finalized, we dedicated our fall meetings to creating the CAB’s foundation, understanding what members and research team expected from participation, and informing members on current data collection process updates. In our first meeting we created a series of polling questions where participants could provide anonymous responses to a series of prompts, specifically “what do you want to get out of being part of the CAB?” and “what expectations do you think CAB members should have for participation?” to guide discussion and to help the research team come up with a list for the CAB members and themselves. We then asked members to provide feedback on these expectations over email/during the next meeting to ensure everyone was comfortable moving forward. This also entailed ensuring CAB members had decision-making authority on all aspects of the study. However, we must note that we had begun to recruit schools for the needs assessment and had a semi-final data collection method in place given that this study is funded through a larger grant. Thus, the CAB was not able to weigh in on the grant proposal nor initial survey/interview guide design.

Sharing the mixed methods needs assessment survey results with CAB members allowed them to provide nuanced insights that may have been missed or left unexplained in the data. All members provided input on interview guide finalization and subsequent analysis steps. While sharing how the research team coded qualitative interview data, we encouraged CAB members to identify key terms are not end-user friendly so we can make note of construct titles to condense when presenting findings. For example, some of the constructs pertaining to “outer setting” factors (i.e., neighborhood environment, district policies), they suggested changing the name of “local conditions” to “local environment” to better explain factors within the surrounding area that may impact meal implementation. Finally, sharing overarching findings as they arose was extremely helpful so the CAB (who except for 1 member are not trained researchers) could give insights iteratively throughout this longer process. Input and advice from CAB members also let to them sharing their own experiences of the program which strengthened the validity of our coding through enhancing transferability of findings.

### Understand the context

The research team reviewed the existing literature on food insecurity inequities to understand the context of the health issues that members of the greater Philadelphia area face. This allowed our team to understand the relationships between food insecurity, obesity, and other chronic disease inequities, and how these critical issues might drive how schools approach USM implementation. This also gave us a foundational understanding of the target population before discussions with the CAB members and allowed for a collaborative interpretation of initial survey findings and observations.

The February 2024 meeting focused on sharing possible methods for dissemination and understanding what barriers are to consider in dissemination of research findings to various target groups that the CAB felt would benefit most from our research findings. In discussing experiences of the various CAB roles, ranging from research to end-use, we were able to address barriers such as literacy level and potential mistrust toward researchers (due to often not benefitting from research, feeling exploited, and other structural issues documented in the literature)[37,38], which may affect how consumers would interact with our work. As a group, we decided that dissemination should be designed for three different groups: Students/families, school leaders, and the broader Philadelphia community, given the prioritization of local-level dissemination. Depending on the target group, certain delivery channels were deemed appropriate because of the demographics associated with it, for example, providing findings with an accessible readability level to administration of partnered schools to share with their students to respect the trust between the staff and students. For students across the district, social media posts were considered more accessible and more appropriate. In contrast, parents of students may benefit from a town hall following the social media posts where they are able to discuss the findings and provide input on recommendations moving forward. The research team and CAB members felt that community members involved in advocating for food security or child/adolescent health may appreciate a presentation and discussion with CAB or student representatives that can advocate for the research conducted.

### Confirm and co-design product

Using a toolkit developed at the University of Minnesota [39], the research team facilitated the dissemination co-design process between February and April 2024. During the February meeting, members were split into groups to brainstorm dissemination ideas for the three target populations. Students and parent representatives led the student/family social media brainstorming, our nonprofit partners led the community-facing materials brainstorming, and our academic and other student members led the school district report brainstorming; the research team felt these initial groupings would provide strong initial ideas for our dissemination products.

Copies of the dissemination action plan worksheet were distributed to each group to guide discussions on formulating dissemination plans that will be most effective for each group. The toolkit included 5 prompts for discussion including: 1) Identify dissemination goal, 2) describe target audience, 3) key research finding/message to share, 4) what dissemination product type(s) will be most effective at targeting your audience and sharing your message? and 5) describe the final product. Each group took notes during brainstorming discussions and then disseminated via presentation to the CAB and research team for feedback. This guided development of the dissemination plan, described below.

### Develop dissemination plan

After deciding on the products and developing initial drafts (in Canva®) for the CAB to review during the March meeting, the April meeting focused on when and how these materials would be distributed. In addition, we discussed how the timing of local-level dissemination would align with writing and submitting a peer-reviewed manuscript on the needs assessment findings. The CAB and research team agreed that dissemination of research would prioritize the schools and the local community before writing a peer-reviewed manuscript. Following this decision, the group established internal deadlines for finalizing products and set targets for initial development and review of materials.

### Plan for sustainability and evaluate iteratively

For this case study, these two final steps were combined as we approached these in parallel. To support our evaluation of the CAB itself and the degree to which our co-creation of dissemination products is an effective way to engage their collective expertise, we adapted a survey tool that had been used in a longitudinal evaluation of CAB members’ perspectives of involvement and overall impact[3]. The survey assessed five domains: Mission, Commitment, Communication, Respect/Trust, and Teamwork/Balance of Power, using a Likert-Scale response. Example questions include “The board has developed a set of guiding principles that is agreed upon by all members” (Mission) “There is adequate commitment on the part of all participating organizations to maintain an on-going board” (Commitment). All items are reported in the results section. Responses to the survey were scored on a 1–4 scale whereby 1 = strongly disagree and 4 = strongly agree.

To provide meaningful data on CAB members’ perceptions of the board’s impact and areas for improvement, the research team added several free-response questions such as “Why did you initially join the CAB?” and “What are ways the CAB could improve over the next 6 months.” Two rounds of the survey were administered: baseline in November 2023, shortly after the first two meetings and one in April 2024 as a follow-up. Finally, we have begun to evaluate the initial impact of dissemination by 1) tracking website and social media activity and 2) documenting the usage and sharing of documents we disseminated via email and our website. This evaluation is in the early stages; our goal is to track document downloads and integration of our work into other products such as research briefs, websites, and social media. We hope to report these metrics in the coming years.

### Data analysis

The goal of data analysis was to track actions taken to establish and maintain engagement during monthly meetings while assessing the perceived efficacy of CAB participation. This comprehensive approach ensures a nuanced exploration of both quantitative and qualitative data, contributing to the full evaluation of the school meal program and the experience of community engagement using a Community Advisory Board. The survey data were analyzed descriptively. Quantitative scoring data were analyzed to generate means and SD for the baseline and follow-up timepoints. For the qualitative data collected from the open-ended survey responses, content analysis was conducted given there are not enough different data points to ensure robust thematic analysis. Minutes and documents from each monthly meeting were invaluable in documenting the progress of the CAB and main items accomplished over the course of the year. These helped form the narrative of the results section and accompanied the data presented to address study aims.

## Results

Below we provide a summary of the results from this case study according to each step of the D4DS model. Some steps may be combined where data collection efforts accomplished both steps. In addition, Empathize and Outline the Problem and Understand the Context are not associated with empirical data sources but were important foundational discussions for the subsequent steps; therefore, these are not discussed separately in the results section.

**Identify Partners**

Table 2 provides demographic information for the CAB members including role, gender, self-selected race and ethnicity, age, education level, employment status, and household income. Most CAB members were female and identified as White, with at least a high school level of education. The majority were employed in a full-time position with a household income above $50,000, which is just below the median household income threshold for Philadelphia ($60,698), based on Census data[40].

**Table 2.** Demographic data for community advisory board members

Variable	Frequency	%
Role on the CAB		
K-12 student representative	2	25%
Parent representative	2	25%
Non-profit organization representatives *	3	38%
University representatives	1	13%
Gender		
Male	4	50%
Female	4	50%
Race/Ethnicity		
White	5	63%
Middle Eastern or North African	1	13%
Asian	1	13%
Black or African American	1	13%
Age		
17 or younger	1	13%
18–20	1	13%
21–29	2	25%
30–39	1	13%
40–49	3	38%
Education		
Some high school	1	13%
High school diploma	1	13%
One or more years of college	0	0%
Bachelor’s degree	3	38%
Master’s degree	2	25%
Doctoral-level degree	1	13%
Employment Status*		
Employed and working 1–39 hours/week	1	13%
Employed and working 40 or more hours/week	5	63%
Not employed and not looking for work	2	25%
Household Income*		
$40,000 – $49,999	1	13%
$50,000 – $59,999	1	13%
$70,000 – $79,999	1	13%
$100,000 or more	5	63%

Note: *Nonprofit organizations are Philadelphia-area organizations including 1) a center for university-community partnerships, 2) a community development financial institution, and 3) the food insecurity office within the city of Philadelphia.

### Confirm and co-design product

The completed dissemination plan worksheets can be found in the Appendix. For the dissemination products aimed at community members, the CAB and research team decided to host an in-person or virtual online seminar with a policy brief to share highlighted findings. This could include CAB members speaking along with the research team and open discussion with interested partners. Members of the CAB expressed that all data should be accessible to the greater public to provide evidence to advocate for policies or new programs that could benefit students and neighborhoods moving forward. For students, the student and parent representatives concluded that social media posts were the best way to share findings but also holding a town hall for parents, students, and community members who may still be interested in providing input on the school meals program and possibly providing recommendations for next steps in the implementation research. Due to time constraints, we agreed that developing a social media toolkit for our networked organizations would have a significantly greater impact as they already have their platforms established.

The dissemination product for participating schools was to provide a brief two-page report or infographic of findings with eye-catching visuals. The content would be provided to principals or appointed contacts in each school with a presentation deck to share with their students and teachers/staff in the school. The focus was to inform the schools of the primary findings but also encourage their participation in the following aims of the overarching implementation mapping study.

### Develop dissemination plan

The dissemination plan entailed 1) iteratively creating dissemination products, 2) getting feedback from CAB members, 3) refining and finalizing products, and 4) sharing via new and established networks. Collectively, the CAB and research team chose to focus on developing brief reports for the school district and participating schools, a research brief and presentation slide deck for Philadelphia area organizations, and social media posts (and developing accounts) for students and families. The team also decided that developing a website to house all dissemination products was an essential step so that everything was housed in one place. The first product we created was the District Report (Appendix) which was a brief infographic-style report that highlighted overarching findings and next steps for the 5-year project, in addition to ways that people can get involved. Our colleagues at the district were asked to share this among their networks to raise awareness of the study. Following the district-wide report, individual school-level reports were created following a similar structure with tailored results for each school context. Reports were distributed by the study lead to the principals of each school who were asked to share it among their school community through ClassDojo (education management system) or other channels.

After initial reports were created, we developed a comprehensive presentation slide deck (Appendix) that was presented by the study team to a community-based organization with the goal of reaching the broader Philadelphia community. This slide deck was then used in presentations to the Temple University community and by the study lead at national/international conferences with the goal of reaching higher education audiences. Our team then developed a “research brief” – style document for the community member audience (Appendix 4) which highlighted similar findings to the school district report but focused more on giving context for the study for the naïve reader, with important data on our sample and small graphics that illustrate some of the high-level findings, without the obligation to share exact numbers and percentages as with the school- and district-level reports. We also described the CAB and how members of the local community can get involved in the work to enhance equitable partnerships.

Finally, for students and parents as well as the broader Philadelphia community, our team created a website [41] and social media accounts (Instagram and LinkedIn) over the summer of 2024. Guided by a social media communications plan, the goal was that the website and social media can enhance dissemination efforts beyond the peer-reviewed articles commonly shared by researchers and that our data can become more accessible to those affected by our research. As of November 2024, we have set up a Google Analytics account to track website hits and usage, including how many times project pages were viewed (1.7K views as of April 2025).

### Plan for sustainability and evaluate iteratively

The survey results from baseline to follow-up are presented in Table 3 showing mean values for each question on a 1–5 scale (Strongly Disagree to Strongly Agree) and then collapsed means for each domain. All 8 members of the CAB completed baseline and follow-up surveys. Most constructs and items were ranked more positively (i.e., more people “agreeing” with statements) in fall 2024 compared to spring 2024, with the exception of two questions from the Mission construct: “A written version of the guiding principles is accessible to all group members” and “My participation on the CAB is valuable to the organization I represent” were ranked worse in the follow-up survey. It should be noted that one member changed positions to a less community advocacy-focused organization, and they were the only person to score lower on this item. The other exception was the Teamwork/Balance of Power construct in which scores slightly decreased among most questions in the follow-up survey.

**Table 3.** Survey responses from baseline (October 2023) to follow-up (March 2024)

Domains	Questions	Baseline	Follow- Up	Baseline Avg	Follow- Up Avg	Change
Mission	The board has developed a set of guiding principles that is agreed upon by all members	3.67	3.71	3.74	3.62	−0.12
	A written version of the guiding principles is accessible to all group members.	3.83	3.71			
	My participation on the CAB is valuable to the organization I represent.	3.71	3.43			
Commitment	Members consistently participate in discussion at meetings.	3.67	3.86	3.67	3.81	0.14
	Members follow through on tasks that they agree to perform.	3.67	3.75			
	There is adequate commitment on the part of all participating organizations to maintain an on-going board.	3.67	3.86			
Communication	The board is willing to re-address unsolved issues	3.8	3.86	3.67	3.81	0.15
	I am able to communicate with other CAB members outside of monthly meeting when I would like to,	3.67	3.71			
	I am familiar with the established methods for raising issues within the group.	3.5	3.86			
Respect/Trust	The board is willing to examine topics raised by all members of the group.	3.8	4	3.93	4	0.07
	I am comfortable asking questions during meetings if information is unclear.	4	4			
	I feel that my opinions are respected by other CAB members during meetings.	4	4			
Teamwork/ Balance of Power	Meetings are held at locations that are easily accessible to members.	3.71	3.57	3.77	3.54	−0.23
	Members of the board who have resources (i.e., money, equipment, contacts, expertise) share those resources with the group.	3.86	3.43			
	Members share credit with the whole group when presenting accomplishments of the board.	4	3.57			
	Active members represent diverse organizations within the community.	3.5	3.57			

A high-level summary of findings from open-ended survey responses is displayed in Table 4. Given the small number of CAB members, we did not conduct in-depth qualitative coding and instead displayed findings according to the question they responded to with some relevant quotes and whether these quotes came from the baseline (B) or follow-up (F) survey. In the baseline survey, when asked about joining the CAB, most participants shared their passion for addressing food insecurity and alignment with their professional role. When asked what CAB members hope to achieve, responses related to wanting to be part of a change in policy and to increase student/community member representation in research. We also highlight two of the questions we asked in the follow-up survey about continued involvement and how they want the CAB to improve in the coming months. Participants stated they continue to be involved in the CAB to contribute to and learn from the research process, to build skills in communication, and for fulfillment out of the process. They provided tangible feedback for how the CAB could be improved including increasing/expecting more participation from members, improving communication among the research team and members (i.e., frequency, tasks assigned), and wanting more in-depth research experience and coding practice. Overall, participants shared positive experiences and feedback about the CAB in addition to areas that we as a research team should work toward to improve engagement in the coming years.

**Table 4.** Responses from open-ended survey responses

Questions	Summary of Responses	Relevant Quotes (B = Baseline, *F* = Follow-up survey)
Why did you initially join the CAB? (Asked in baseline survey)	Passionate about addressing food insecurity	“I joined the CAB to increase civic engagement and be involved in another sector of children health.” (B)“I think that all students in Philadelphia having access to free meals could be invaluable if the majority of students and families thought those meals were delicious, nutritious, and filling. Currently that’s not true and I want to support figuring out how we can make that a reality.” (B)
	Alignment with professional interests	“I actively work in examining food systems in Philadelphia and found the CAB to be a strong space to expand my knowledge and share my work/expertise” (B)
What do you hope to achieve through being a part of the CAB? (Asked in baseline survey)	Be part of a positive change in school meals	“To be part of creating a set of recommendations that can be presented to local, state, federal governments and to local institutions with the resources to make positive changes to the school meals program. Especially in ways that can support local Black and Brown growers and food businesses.” (B)“Help with the implementation of a needed improvement to our school-lunches” (B)
	Increase student representation in research	“Learning and networking, to contribute my ideas/opinions on an important topic; to network with other people interested in the topic” (B; student)
Why do you continue to participate in the CAB? (Asked in follow-up survey)	Seeking research experience	“This group continues to impress me with their direction and data analysis. It makes me excited for what’s to come.” (F)“Because I committed to do it and I’m learning more about how qualitative research is conducted.” (F)
	Wanting solutions to improving participation in USM	“The work that the research team is doing is really valuable, and the environment and the team as a whole is so supportive. Working together with others on the board is such a rewarding experience.” (F)
What are ways the CAB could improve over the next 6 months? (Asked in both surveys)	Greater expectations for members during/between meetings	“More involvement/participation from CAB members” (F)“I would like to see a concrete output such as a publication” (F)At the January meeting I would have liked to do another overview of the qualitative coding techniques before diving into the examples. It’s helpful to go back and give a summary of what we had discussed at previous meetings since we’re only meeting 1x/month (F)
	Better communication of study findings	“Communicating what we’re hearing from students, families, and school staff to School Administrators, City Government Officials, and local institutions who might have power/resources to make changes” (B)

## Discussion

The research questions guiding this case study were 1) How can researchers meaningfully build a CAB to enhance implementation research? and 2) How can the D4DS process be utilized in collaboration with a CAB to enhance equitable dissemination of research evidence? We were successful in recruiting and retaining 8 members of the community representing a diverse range of roles to create a collaborative culture and strong foundation in the first year of an ongoing implementation mapping study. The CAB was recruited at the beginning of the study and supported data collection and analysis of the needs assessment (Aim 1); they are now engaging in co-development of Aims 2 and 3 as we are developing implementation strategies with pilot schools and preparing for the trial. Involving them at the beginning of the research process has been instrumental thus far.

One potential challenge when developing a CAB is that the activities and decisions may feel disconnected from the work being conducted in a particular research study, making it difficult to show how a CAB improves the research process or other salient activities[8]. We utilized the D4DS process which is a pragmatic way to facilitate co-creation of dissemination products and plans with community partners[13,15,36,42]. By following these steps and embedding the process within the CAB activities, we were able to educate CAB members on implementation science research and qualitative data collection and analysis. This provided a critical foundation for steps 2, 3, and 4 of the D4DS process which focused primarily on understanding context and co-development of products and a dissemination plan. The principal investigator was trained through the university’s office of Community Engaged Research and Practice on how best to work with and educate community partners prior to its discontinuation. Since CAB members were familiar with the main findings of our study, they could help us frame the highlights of the needs assessment findings to be most salient to specific audiences. We suggest that researchers working in the community could replicate and adapt or refine our process to advance the science of community engagement and enhance equitable dissemination of research.

To plan for sustainability of the CAB and dissemination and to evaluate iteratively, we are just beginning to see the impact of our dissemination efforts and CAB involvement in the local community. Our collective efforts to build a new website and start an Instagram and LinkedIn page are a concrete step in sustaining dissemination and our team has been posting weekly since the beginning of the second year of this study, increasing engagement with our work. Several CAB members have been featured on our posts and supported creation of content to be shared on these platforms. This demonstrates our commitment to sustaining dissemination and expanding on what we accomplished in the first year.

Starting with evaluating the impact of the CAB from the perspectives of our members felt most appropriate, and the feedback received has already begun to be implemented. For example, members wanted more opportunities to be authors on products, and we are currently writing a practice-focused commentary on the CAB and lessons learned from the first year in which all members have the option to be an author. In addition, the online meetings were helpful but not as impactful for improving collaboration; therefore, for our second year, we decided to hold in-person meetings (with hybrid video call option) but fewer of these meetings to make the best use of time and have key milestones where CAB members are in the same room.

Finally, to improve accountability for members, we are revisiting expectations for the second year and trying to be more explicit with what each member should be contributing to the board and the research process. We hope this will build on the foundation we built in year one and advance our efforts to have a true partnership with community members to enhance the impact of our work, attaining all principles of community partnered research[7]. These steps are aligned with a recent report from the Centers for Disease Control on community engagement[33], and we note certain areas for improvement such as shared governance, which is a critical step for us to consider as we deepen the academic-community partnership.

### Strengths

One key strength of this study was our commitment to student engagement and representation. There are examples of CABs effectively harnessing the input of student voices to improve their experiences [12,43]. A scoping review and other studies revealed that student participation not only enhances the efficacy of interventions but also has a positive impact on students’ motivation, engagement, and identity development[44,45]. We also see great strength in co-creating a dissemination plan and products which demonstrated a focus on local-level dissemination before peer-reviewed articles. This seemed to help 1) build trust among CAB members and the community and 2) improve the research team’s ability to translate research findings to the local community.

## Limitations

With only one year of data collection, we cannot say with certainty whether the CAB and the current structure is sustainable. Future evaluation efforts are focused on sustainability with community engagement to avoid exploitation of community members. The lack of engagement of CAB members while writing the research grant should also be noted, although it was not possible to provide compensation to members without a grant. This highlights challenges with university-community collaborations and warrant systems-level change to support community partnered research at the university level. Further, our findings are limited to one geographic area of a high-income country, with many of our members earning above the median income level, so our approach may not be generalizable to other regions and countries. Finally, representation was diverse across racial and ethnic groups and education level but given that the population of Philadelphia identifies as 45% Black/African American, we seek to improve representation of Black individuals on this CAB in the subsequent years by focusing recruitment through our website and social media channels.

## Conclusion

This study documented the process of developing and maintaining a CAB within a 5-year implementation mapping study to enhance the equitable impact of USM and engaging in the D4DS process to promote dissemination of research findings. We believe this greatly improved our research and future aspects of the implementation mapping process with SDP by learning from our CAB members throughout the first year. Our approach serves as a guide for researchers and advocacy groups to integrate community expertise, especially for addressing complex issues such food insecurity and nutrition policy. We encourage scholars to view community engagement as a science requiring adaptation and course corrections, to better engage end-users of research and bridge the gap between research and practice.

## Supporting information

10.1017/cts.2025.10065.sm001 Mcloughlin et al. supplementary material Mcloughlin et al. supplementary material

## Data Availability

Data associated with this manuscript can be requested from the corresponding author.

## Supplementary material

The supplementary material for this article can be found at https://doi.org/10.1017/cts.2025.10065.
