# Supplementary material for: Designing for dissemination through community advisory board engagement in an implementation mapping process: A case study
Source: J Clin Transl Sci. 2025 Jun 9;9(1):e138. doi: 10.1017/cts.2025.10065 (PMC12260986; doi:10.1017/cts.2025.10065)
Supplement: Mcloughlin et al. supplementary material [file S2059866125100654sup001.pdf]

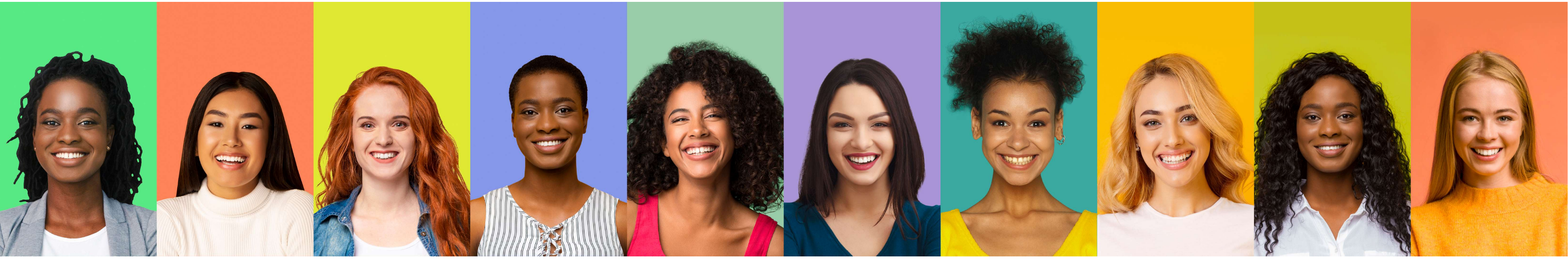

# 3A Dissemination Action Plan Worksheet - Use this worksheet to plan and create your dissemination product(s).

| | |
|---|---|
| **Dissemination Goal:**<br>Copy from Step 3 | **Example:** Raise awareness and educate the Twin Cities Hmong population on COVID-19<br><br>To offer the policy/statistics for community partners (NGOS, non-profits) to use for RFPs and opportunities to fill need and provide resources.<br><br>Increased awareness of food issues in offering of research |
| **Target Audience:**<br>Copy from Step 3 | **Example:** Hmong population in the Twin Cities including multiple generations of Hmong families, especially elders<br><br>Community partners, NGOs non-profits and business partners in Philadelphia. |
| **Key Research Finding/Message to Share:**<br>Copy from Step 3 | **Example:** General information on COVID-19 and how they can prevent the spread<br><br>-what are the challenges around school meals?<br>-what are the current needs and "pressure points"<br>-emphasizing the fact that the one size fits all model doesn't work -- need for different vendors and putting responsibility and resources in smaller sections (regionally), show examples of where there has been success in the school meals program |

| | |
|---|---|
| ***What*** **dissemination product type(s) will be most effective at targeting your audience and sharing your message?**<br><br>**Circle each product type you will create and complete the related column. (example)**<br><br>**Choose which product type you want to use:** | **Product Type Examples:**<br>**Example:** Video<br><br>- [x] Policy brief<br>- [ ] Fact sheet<br>- [x] PowerPoint slide presentation<br>- [ ] Poster presentation<br>- [ ] Infographic<br>- [ ] Promotional products & giveaways (e.g. stickers, magnets, notepads etc.)<br>- [ ] Papers & reports (non-peer reviewed)<br>- [ ] Journal articles<br>- [ ] Video<br>- [ ] Adapted cultural performances & dance<br>- [ ] Skit<br>- [ ] Songs<br>- [ ] Education curriculum or training<br>- [ ] Podcast<br>- [ ] Interview script/talking points<br>- [ ] Social media (e.g., Twitter, Facebook, Instagram, TikTok, etc.)<br>- [ ] Other<br><br>webinar |
| **Final Product** | **Example:** We will make a culturally specific video with information on COVID-19 and how to prevent spread.<br><br>show highlighted findings, have representative (ie, students or parents),<br>Webinar or in-person event<br>talk about current policies, experiences, hold open discussions with members about their experiences servicing students<br>could seek a funder for co-pilots or programs to bring to schools, focus on nutritional value, equity in city schools, and how it affects other outcomes like academic performance |

| | |
|---|---|
| ***Who* should share/deliver/promote your final product?**<br><br>**Identify key messengers for each product.**<br><br>**(Examples: community leaders, students, politicians, community organizations, teachers, patients, researchers, doctors, etc.)** | **Example:** Our video will be shared by community leaders and young people in the community.<br><br>Temple CORE, CAB members promote to their network, orgs that filled out interest form in the beginning of forming CAB,<br><br>CAB members can speak with research team<br><br>CHOP: grant for food<br>YEAH<br>Feeding Philly<br>PHENND<br>SNAP-Ed<br>PDEdu<br>New school board members |
| ***How* will your final product be shared?**<br><br>**(Examples: share videos via email, websites; share performances at conference or through community theater)** | **Example:** To reach older generations, we will share the video through community leaders at community events and centers. To reach younger generations, we will share the video on Social Media platforms.<br><br>CAB members share to their network |

| | |
|---|---|
| ***When*** **will your final product(s) be shared?**<br><br>**Are there specific dates or events that are relevant or important to your audience(s)?**<br><br>**(Examples: dates, events, etc.)**<br><br>***Where*** **will your final product(s) be shared?**<br><br>**(Examples: school board meetings, community health fairs, conferences, etc.)** | **Example:** The video will be shared at the next community gathering on April 12. We will launch the video on Social Media (Facebook, Twitter, SnapChat, Instagram) on April 12 also. The video will be available on YouTube and shared with Hmong news channels (e.g., Hmong TV). |

# School Meals at The School District of Philadelphia

**What do the students, parents, and staff at the school district think about school meals?**

## Who did we talk to?

We learned from 193 participants across 8 schools (6 elementary-middle, 2 high) in the district. Participants were varied in terms of race, ethnicity, and we had an equal distribution of roles.

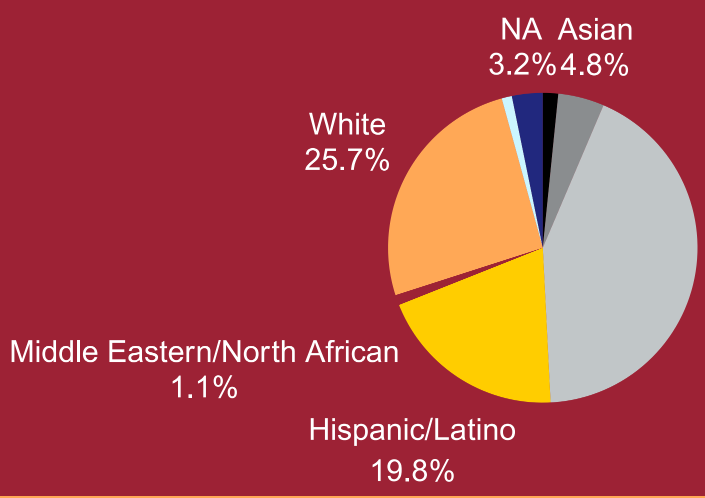


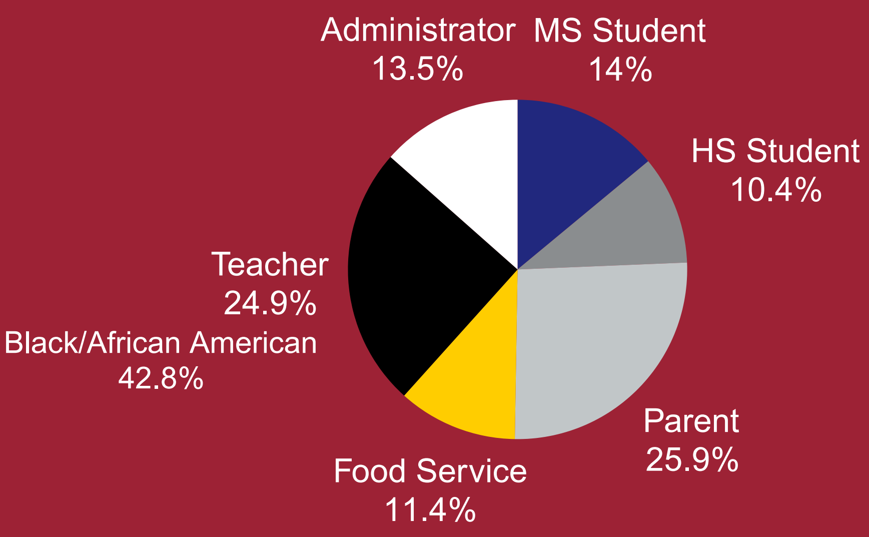


## What did we find?

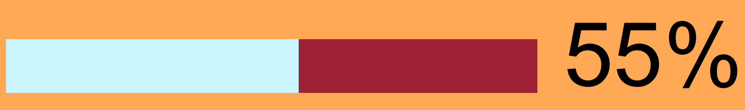


Of students feel that the school food is healthy (answered either “agree” or “strongly agree”)

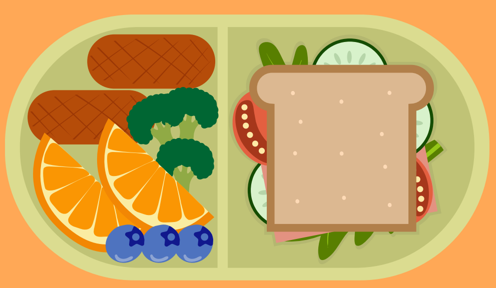

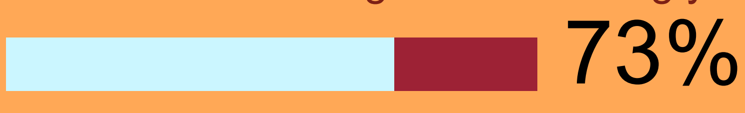


Of students feel school meals helps students struggling with hunger (answered either “agree” or “strongly agree”)

**‘I feel like they put like other stuff on the plate that we don't eat instead of just giving us more of things we like.’ (MS Student)**

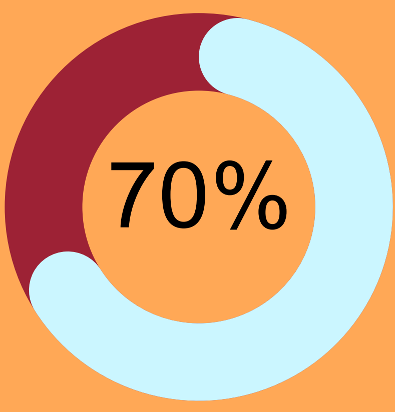


Students feel that they do **not** have a say in the foods included in the program

Of food service responded that complying with this program **somewhat** requires more work than what can be accomplished with current resources available to their school

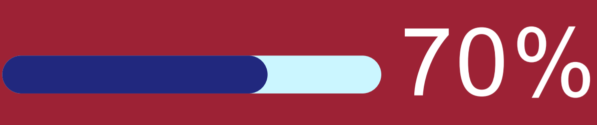


**8 out of 17** administrators believe the school meals program takes the culture of the local area into account

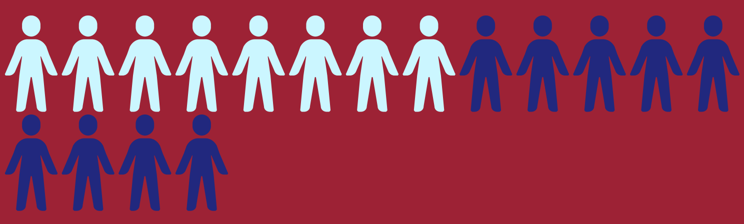

Of parents believe the school meals program takes their beliefs/interested into account

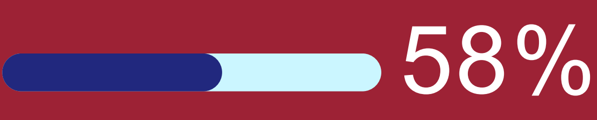


‘The benefits of free lunch and free breakfast is it comes in handy because a lot of parents right now are, you know, living under their needs, you know, living paycheck to paycheck. Sometimes parents don't got, you know, food in their fridge to feed their kids. So being able to go to school and get two free meals is important.’
(Parent)

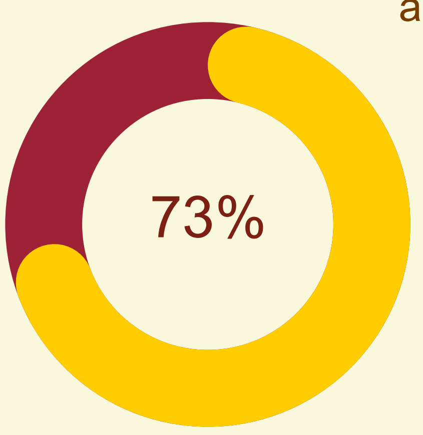


Of parents believe the program somewhat/really helps students struggling with food insecurity

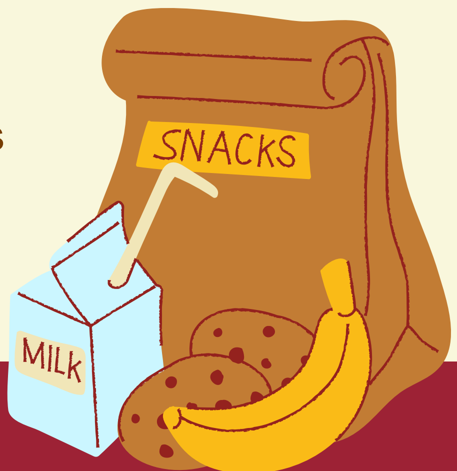

# Most popular focus areas from the district

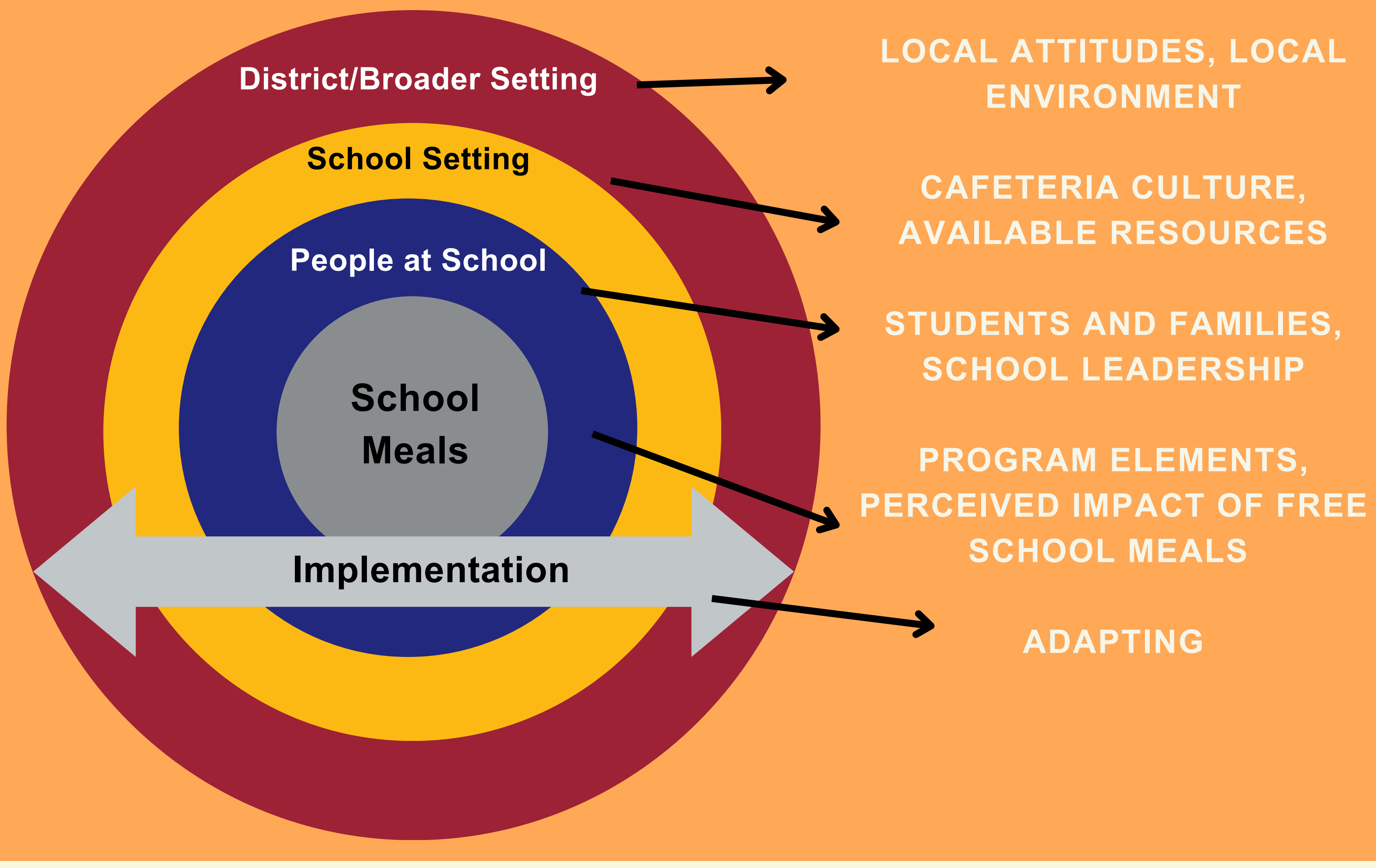


### Insights from Students

'They call it a freebie. Yeah. It's like, oh, you're getting you're getting the free lunch. You suck.' - Cafeteria Culture

### Insights from Parents

'In the last two years, it's become very inclusive and very like we're serving was what it is the thing falafels and stuff... so I know that the district as a whole it is reflecting a different cultures.' - Local Attitudes

### Insights from Staff

'And I think that today, when they eat it, they're more focused. And clearly, they need it, right? Because they maybe haven't eaten yet. And it just gives them that little boost.' - Perceived Impact of Free Meals

## How we will use these findings?

- We are sharing the findings from this study across the city of Philadelphia, the United States, and internationally to raise awareness of school meals
- We are using these results to design and evaluate changes to implementation of school meals next year

## How can you get involved?

- We have a Community Advisory Board (CAB) and are building a community network listserv to share updates on the project. Want to get involved? Contact us below!

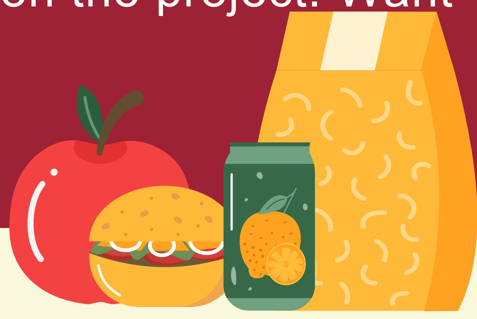

## About us!

We are a research Team at Temple called the **Community-Driven Implementation Science lab.**

**What does this mean?**

We do research that is driven by the needs of the community by partnering with local experts and people who are often not included in research.

We use a range of methods aimed at improving delivery of evidence-based programs to promote health equity

**About our Community Advisory Board**

We led this project in partnership with our Community Advisory Board (CAB). Here are our members and their roles:

**Laura Crandall, Office of Children and Families**
**Dr. Jonathan Deustch, Drexel Food Lab**
**Irene Hong, Feeding Philly/Student**
**Hillary Kane, PHENND/Parent**
**Jacob Kurtz, Reinvestment Fund/Teacher**
**Rose Marone, Feeding Philly/Student**
**Randy Persaud, Jefferson Graduate Student**
**Sultan Raheem, Parent representative**

COMMUNITY-DRIVEN IMPLEMENTATION LAB
EMPOWERING CHANGE IN THE COMMUNITY

**Contact us! Scan the QR code to send an email to our team**

**Our funders!**

NIH National Heart, Lung, and Blood Institute

# Universal School Meals in Philadelphia: Findings from a Mixed Methods Needs Assessment

Dr. Gabriella McLoughlin
Molly Kerstetter
Yerusalem Yohannes
Community-Driven Implementation Science
Temple University

COMMUNITY-DRIVEN IMPLEMENTATION LAB
EMPOWERING CHANGE IN THE COMMUNITY

NIH
National Heart, Lung, and Blood Institute

# Meet the Team

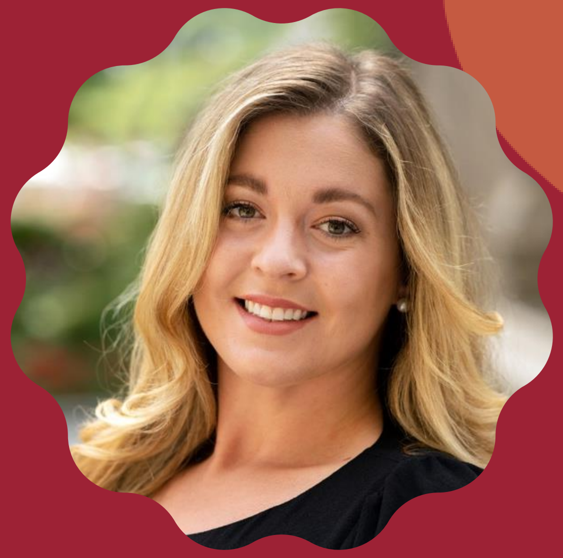

DR. GABRIELLA MCLOUGHLIN
Principal Investigator

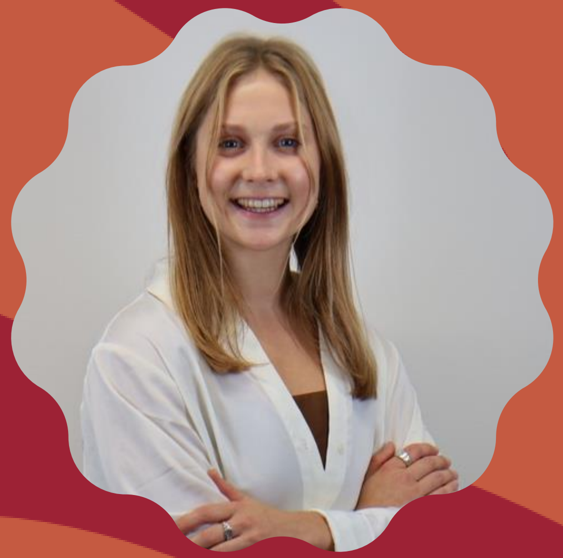

MOLLY KERSTETTER
Research Coordinator

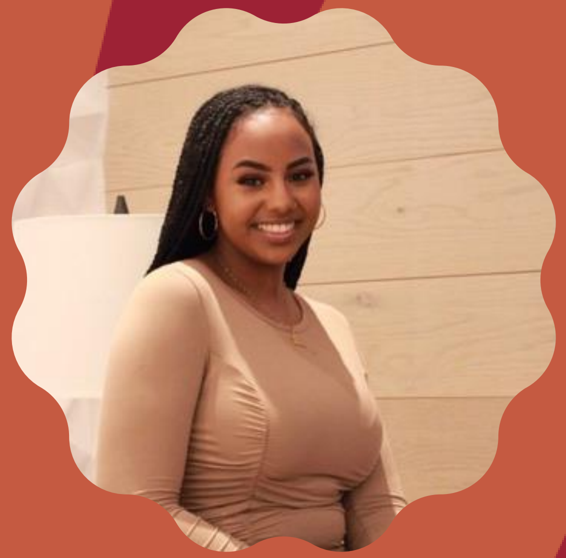

YERUSALEM YOHANNES
Research Coordinator

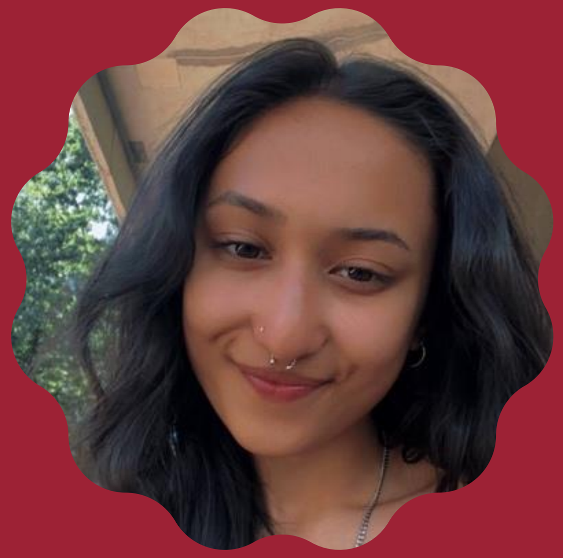

GARIMA ADHIKARI
Research Assistant

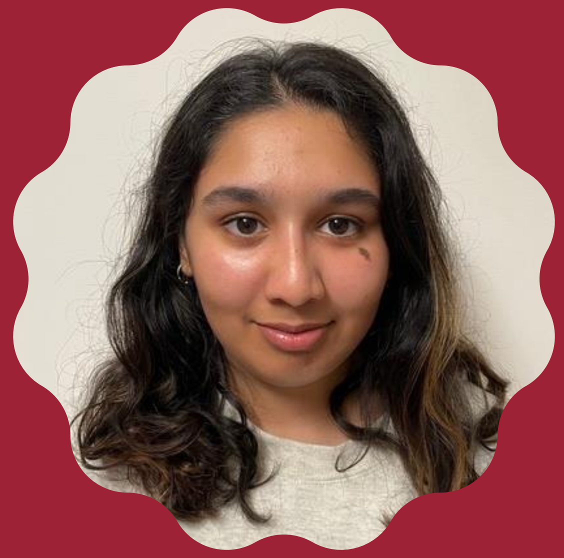

DIVYA KULKARNI
Research Assistant

# Why are we doing this study?

Food Insecurity is rising in Philadelphia

The Community Eligibility Provision allows **ALL** students regardless of financial status to receive both free breakfast and lunch

**Only half of students are eating the meals and we want to know how we can fix this**

# Our Study's Process: Implementation Mapping

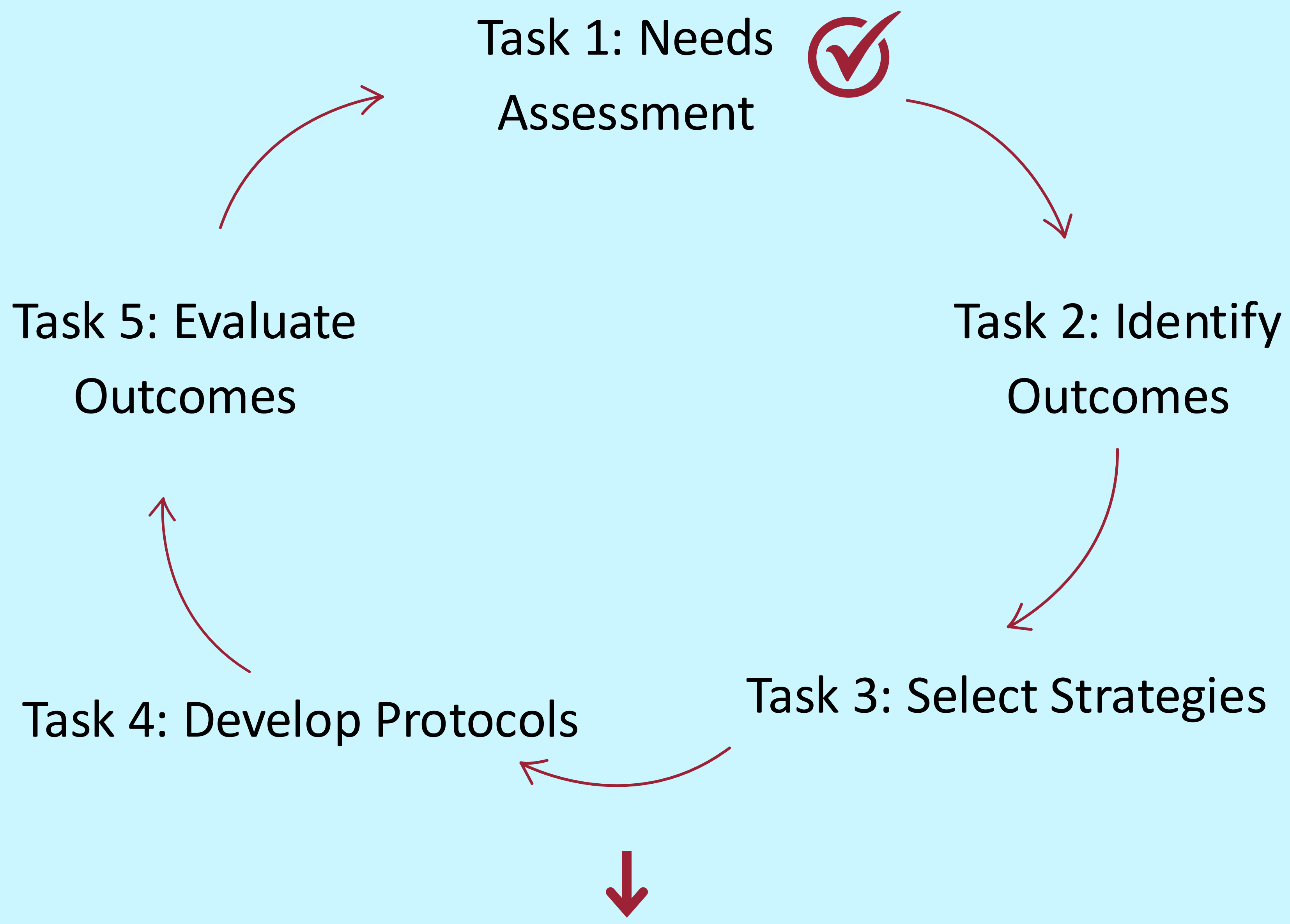


Improved USM program to ease access to free healthy meals

# Overview of Sample

8 schools;

6 elementary-middle (2 satellite service) 2 high schools

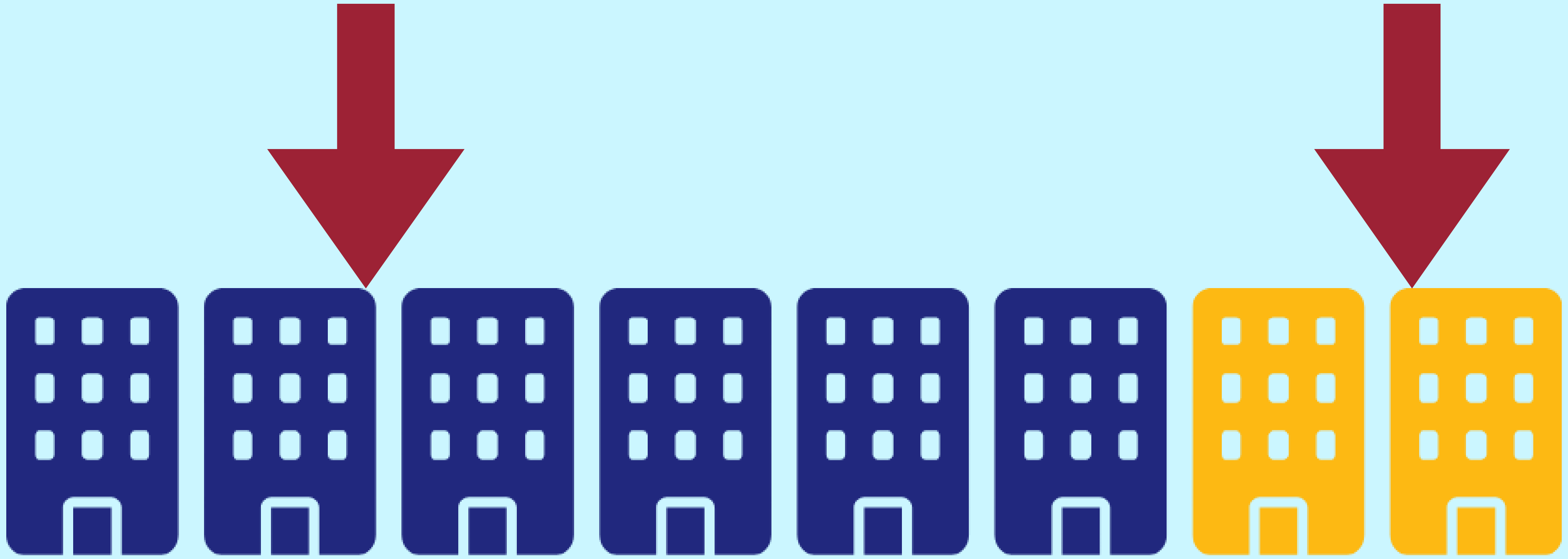

Grouped into High/Low participation based on district averages from Fall of 2023 of 32% participation in breakfast and 48% participation in lunch

# Methods

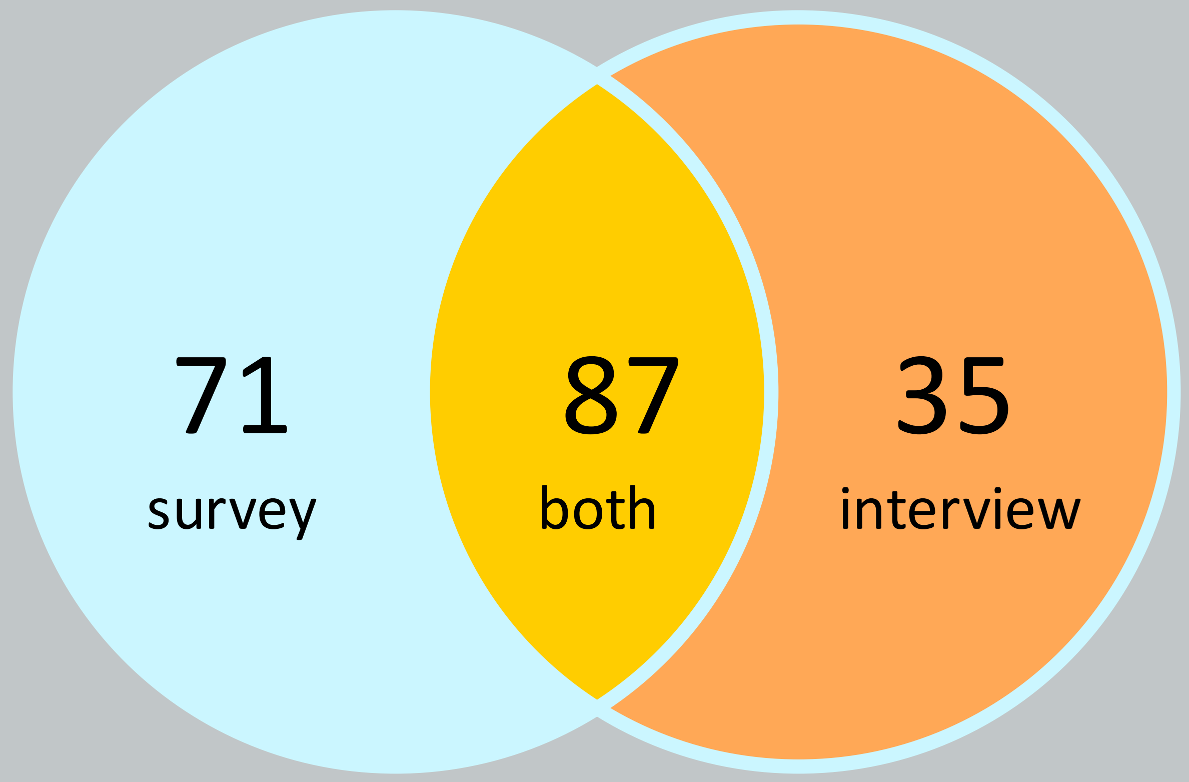


## OBSERVATIONS

We observed breakfast and lunch at least twice at each school

## SURVEYS

We received 158 surveys

## INTERVIEWS

We completed 122 interviews

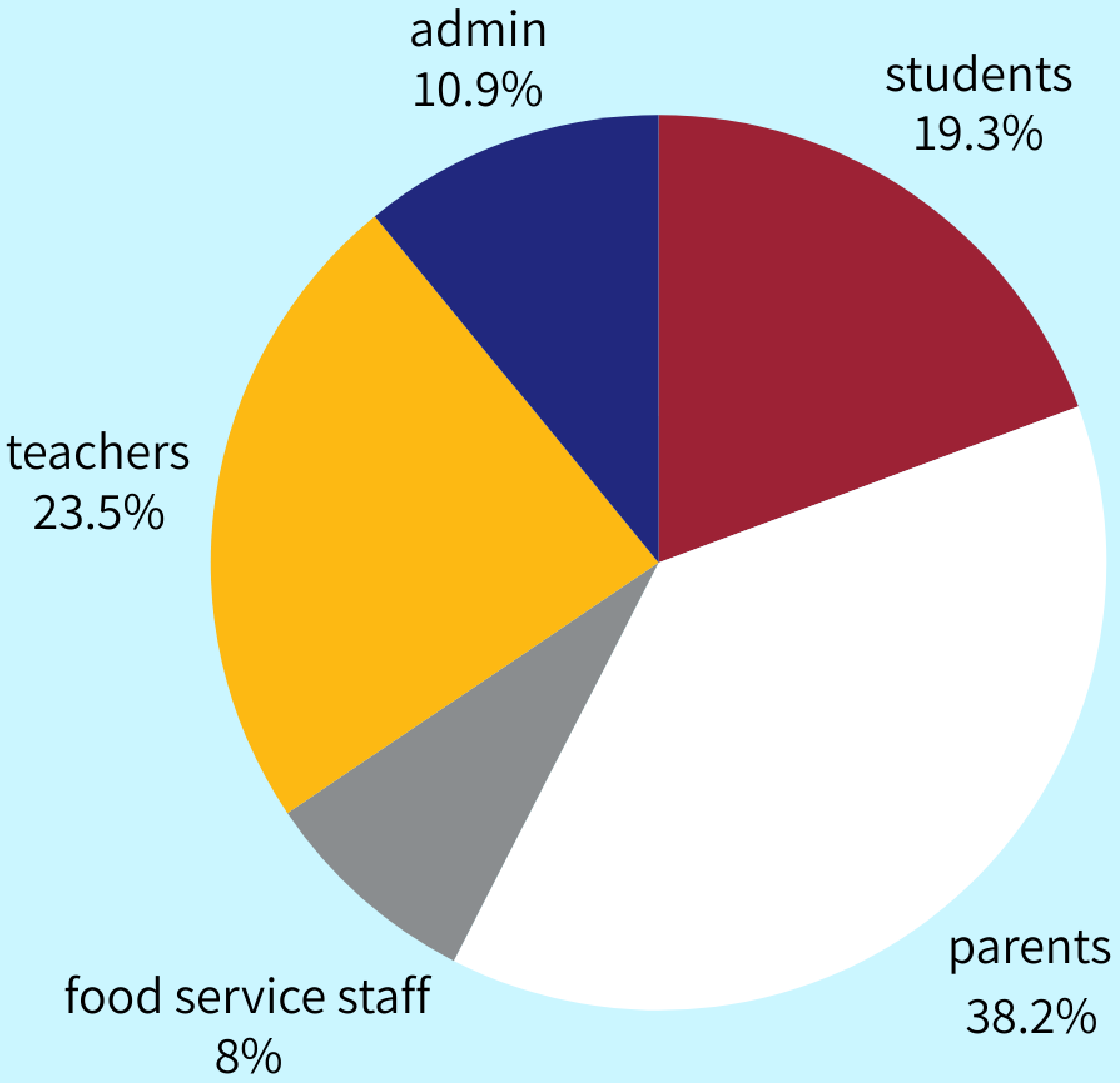


# BREAKDOWN OF SURVEY PARTICIPANT ROLES

# BREAKDOWN OF INTERVIEW PARTICIPANTS

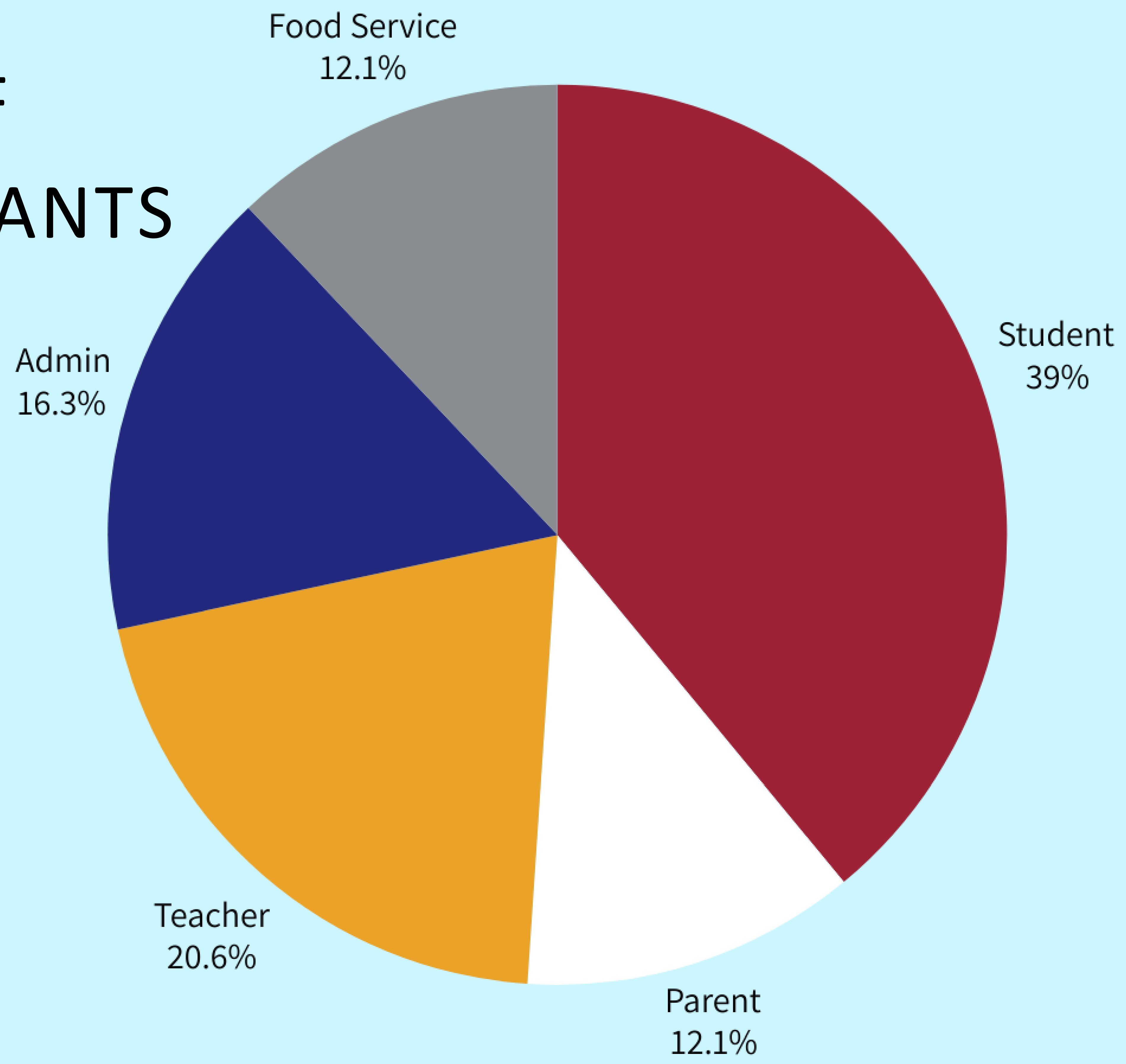

# RACIAL/ETHNIC BREAKDOWN OF STUDY SAMPLE

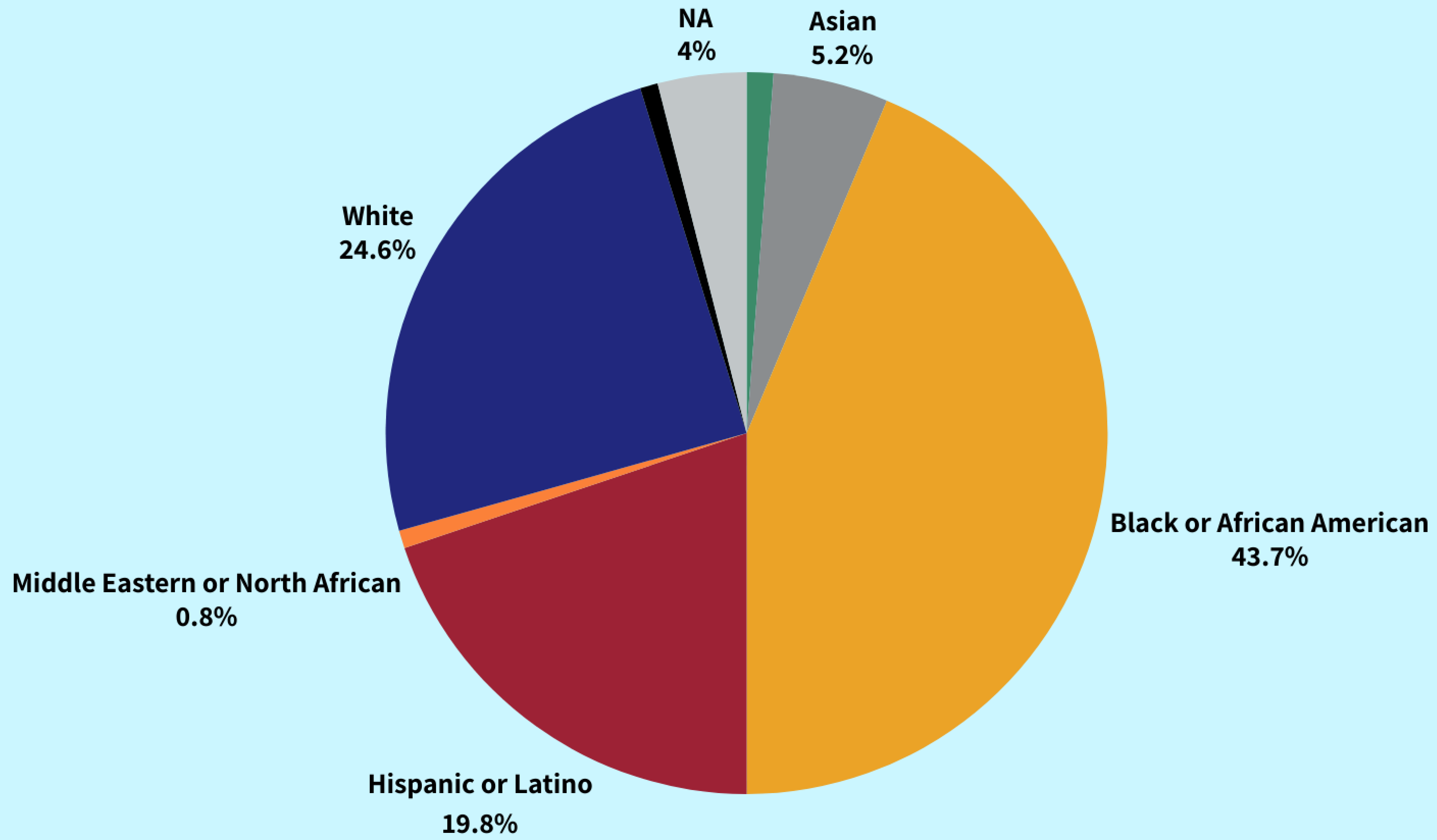

# SURVEY SAMPLE GRADE LEVEL

6 K-8 SCHOOLS (MIDDLE GRADES ONLY)

2 HIGH SCHOOLS

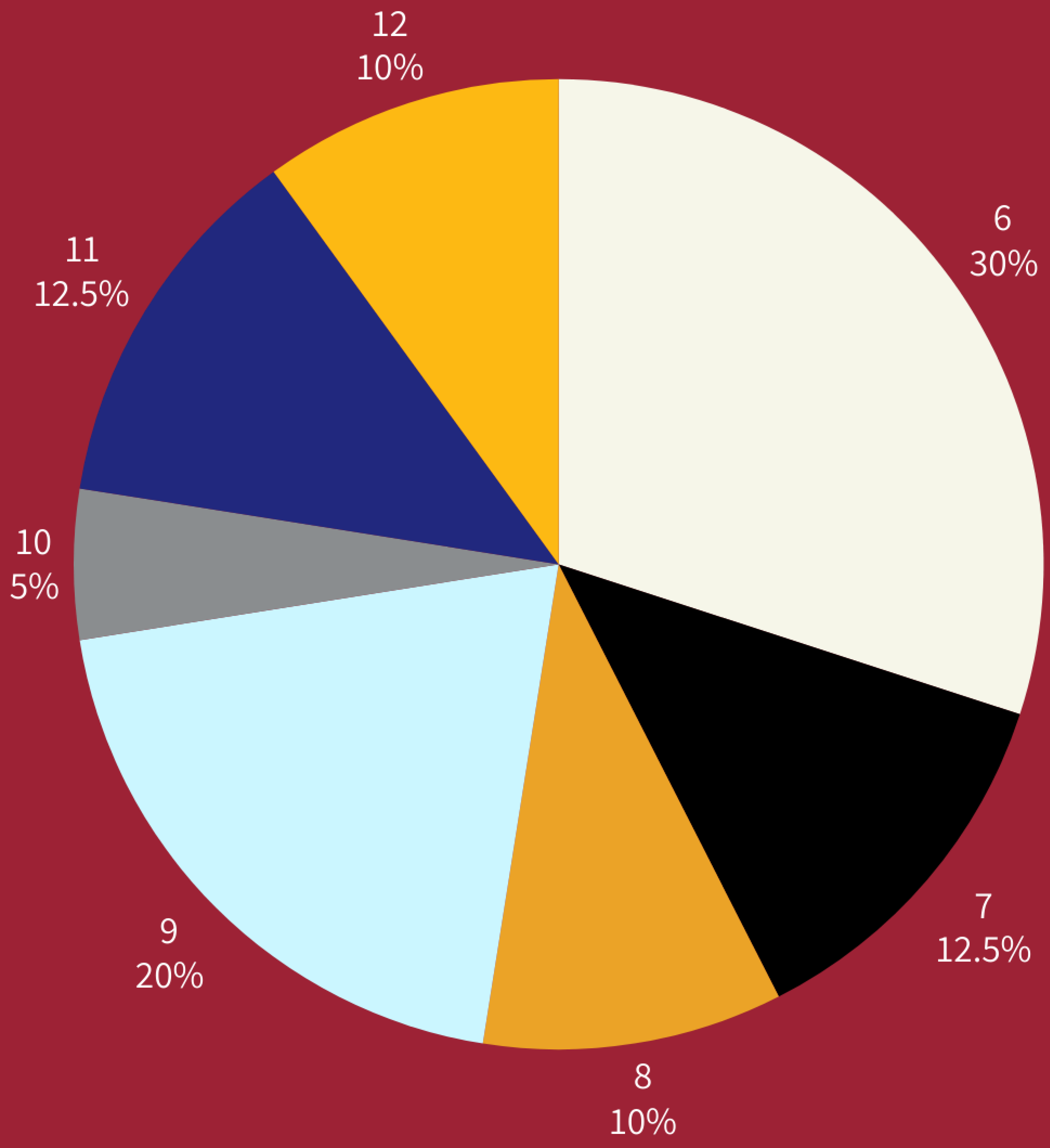

# What is the perceived impact of school meals?

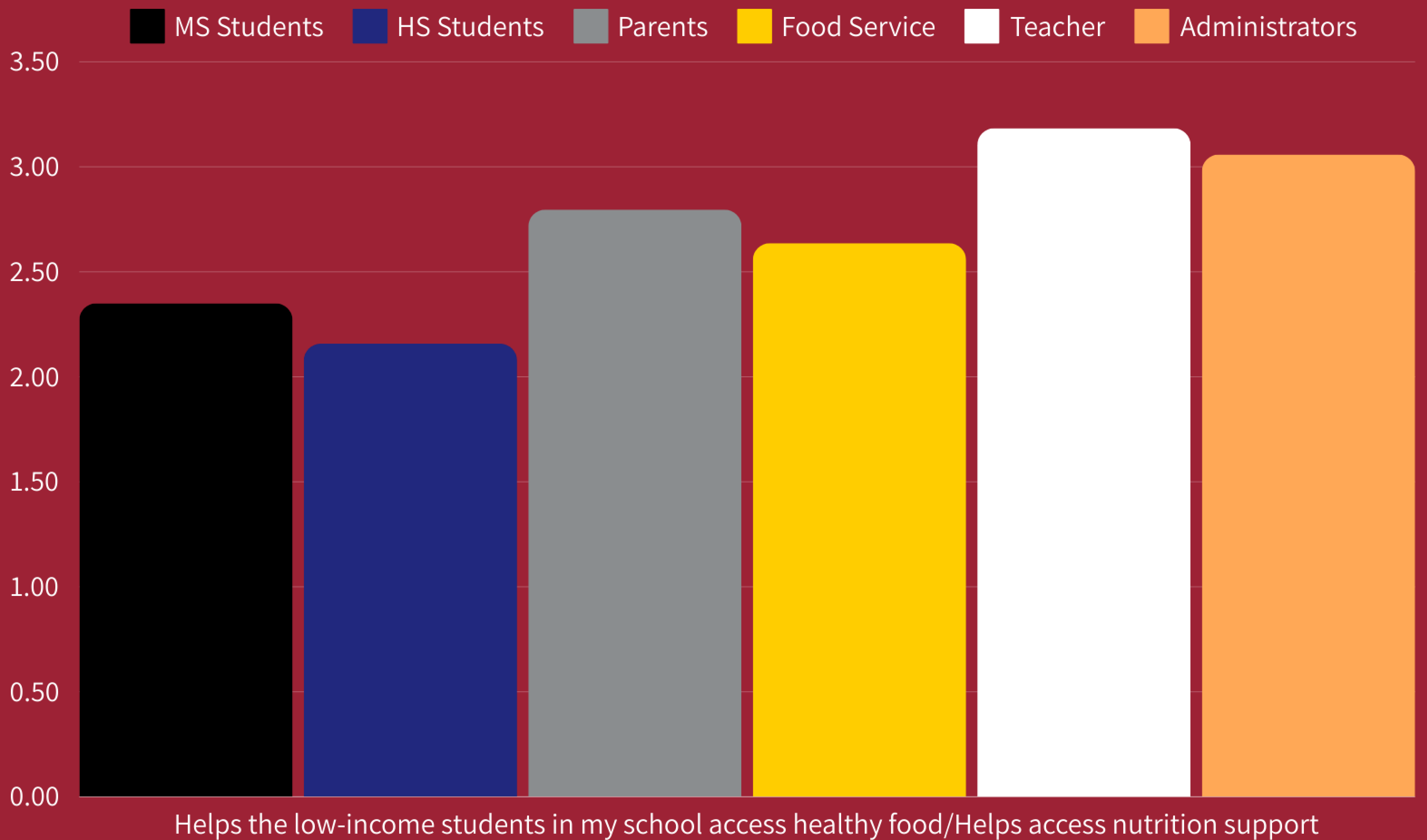

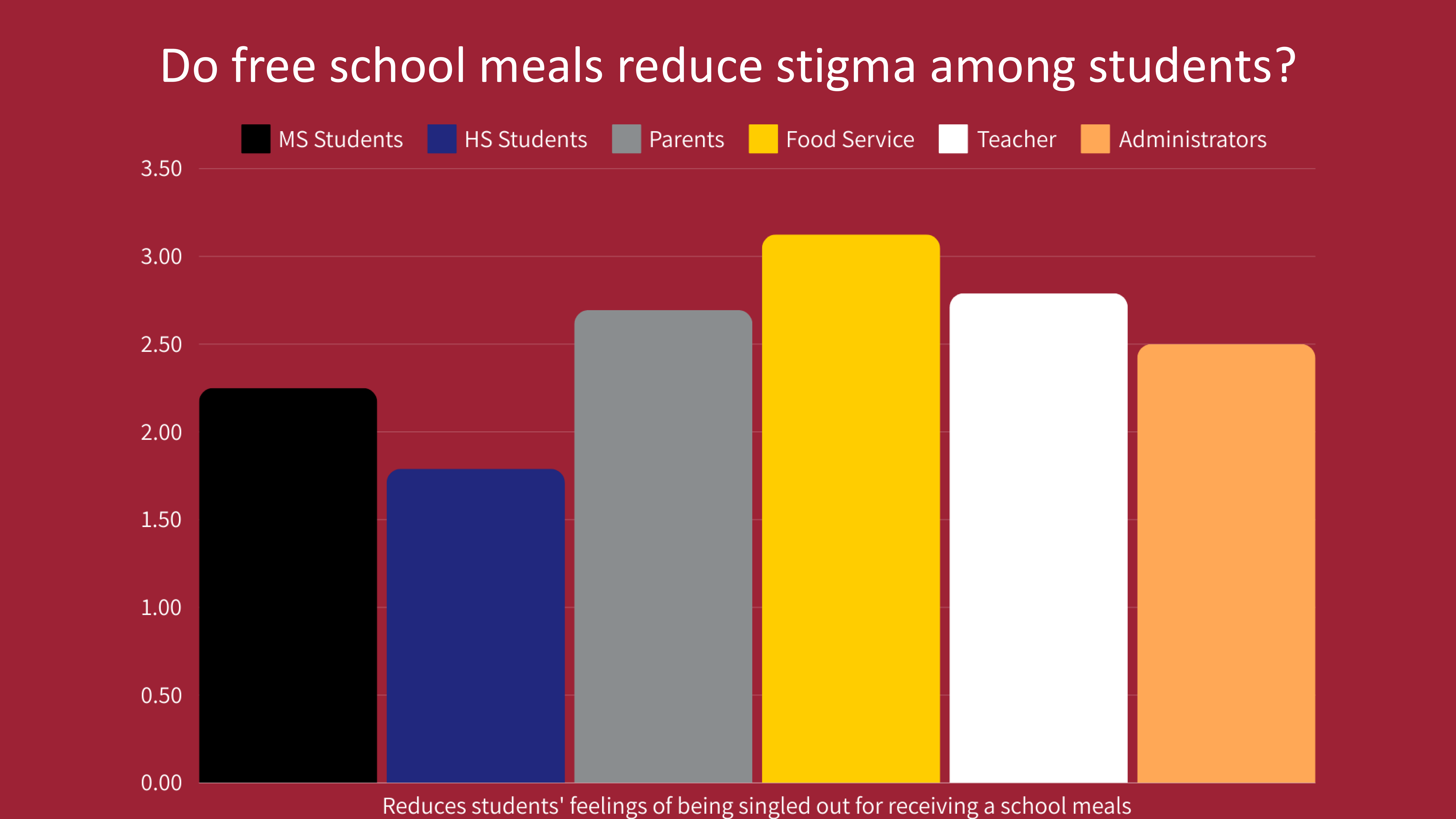
Do free school meals reduce stigma among students?
MS Students
HS Students
Parents
Food Service
Teacher
Administrators
3.50
3.00
2.50
2.00
1.50
1.00
0.50
0.00
Reduces students' feelings of being singled out for receiving a school meals

# Do people feel empowered to give input on school meals?

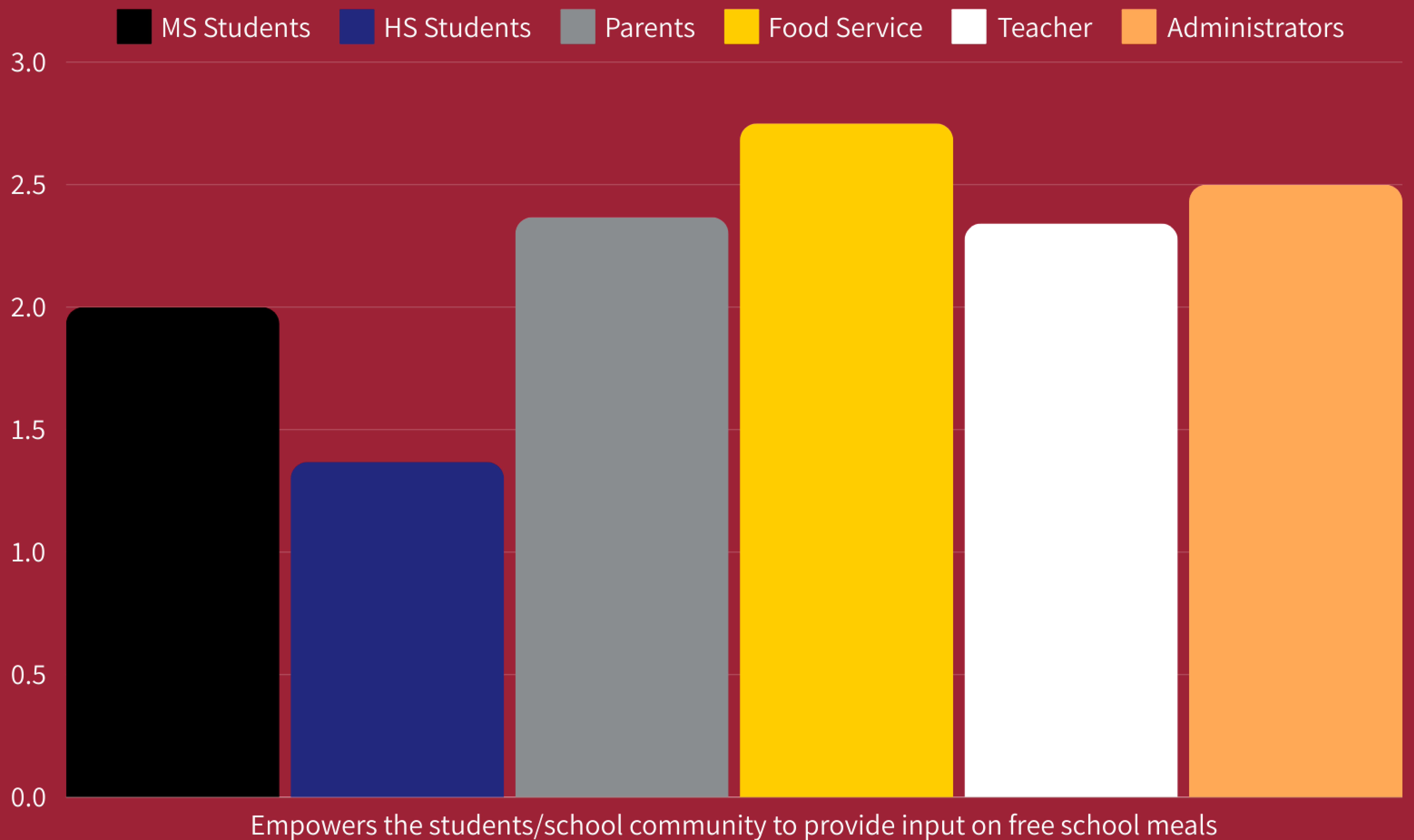

# Snapshot of Major Themes linked to framework

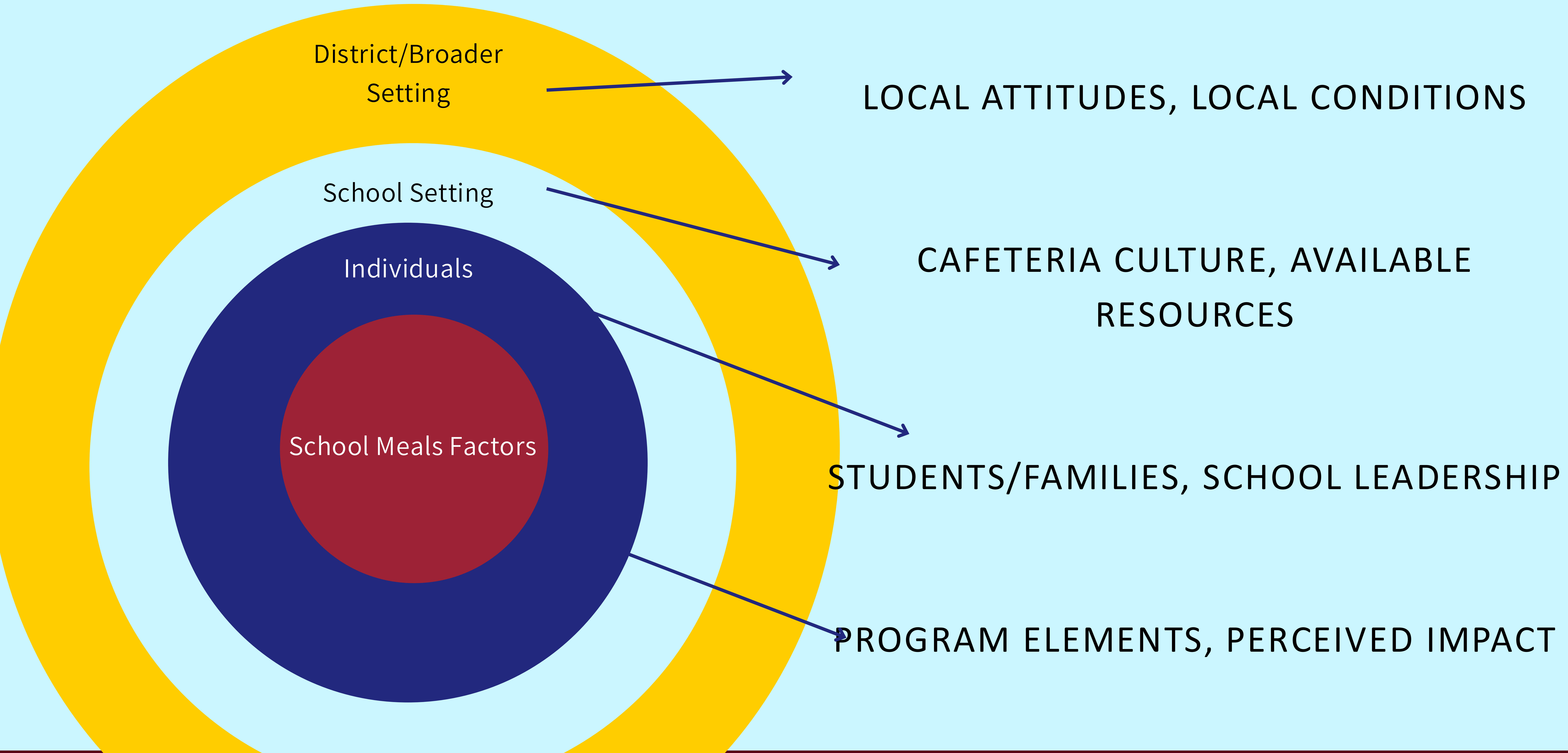

# School Meals Factors: Program Characteristics

+

"Not all the food is bad, or poor quality. You know, there's like **fresh fruit** and **vegetables** and stuff like that. There's food that's fine. **High protein.**" (Principal, sat)

−

"For me, I think of the food quality. I think just to a large extent, if the **food quality were better, the stigma would be less.**" (Principal, sat)

"I mean, I feel like the **food isn't really presentable**. Like, let's say I took a picture and posted on social media. Okay, woah, what is that they're feeding that to you. Yeah. Like, it just **doesn't look appealing** to most kids." (HS Student, FS)

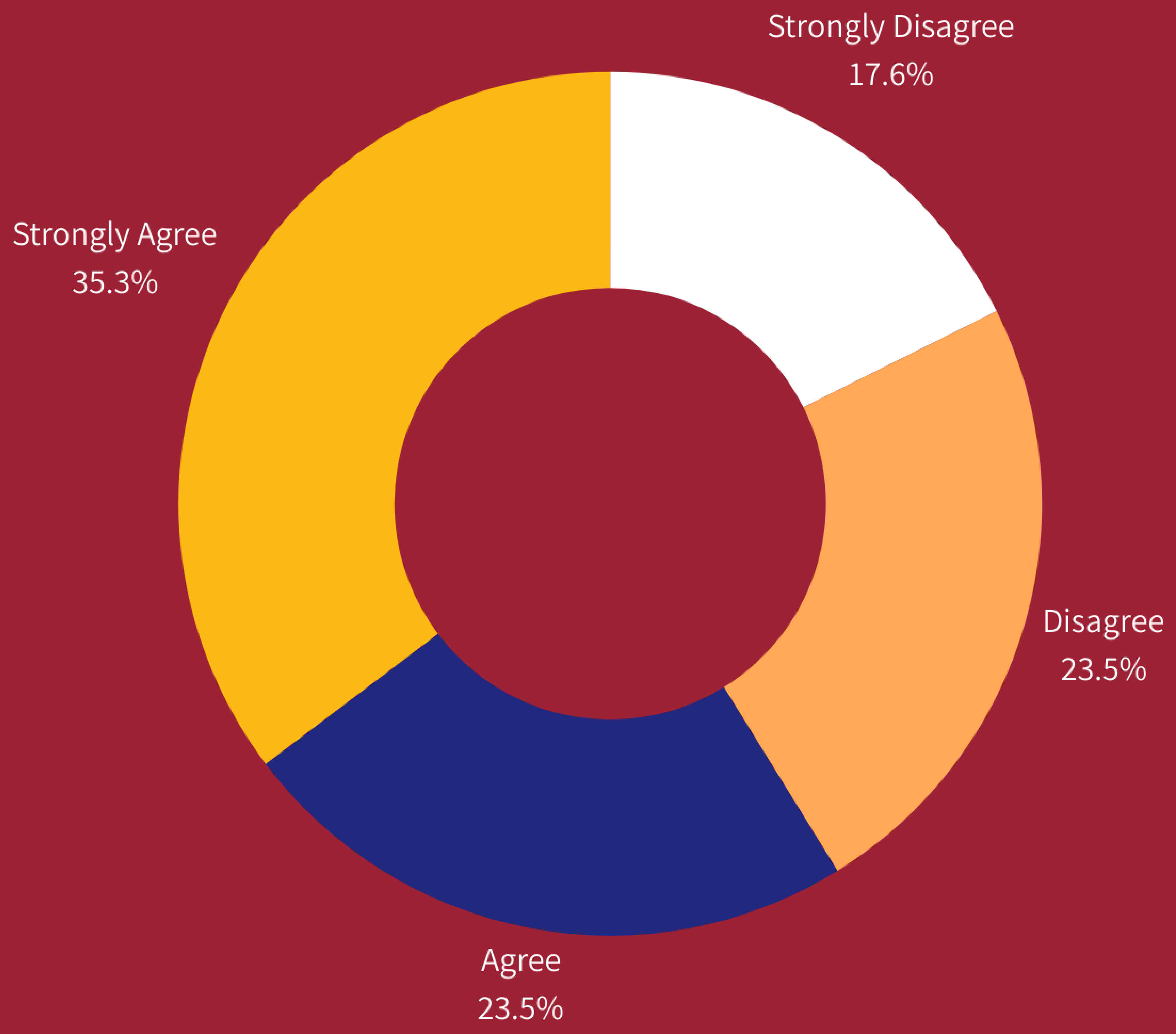


We asked **Admin** if they believed that free school meals increases the availability of higher quality foods to students (compared with foods they can bring from home/buy at corner stores).

# School Meal Factors- Perceived Impact

"And I think that today, when they eat it, they're more focused. And clearly they need it, right? Because they maybe haven't eaten yet. And it just gives them that little boost." (Teacher, FS)

"The benefits of free lunch and free breakfast is it comes in handy because a lot of parents right now are, you know, living under their needs, you know, living paycheck to paycheck. Sometimes parents don't got, you know, food in their fridge to feed their kids. So being able to go to school and get two free meals is important." (Parent, Sat)

We asked students if they think school meals help them be less hungry and ready for learning:

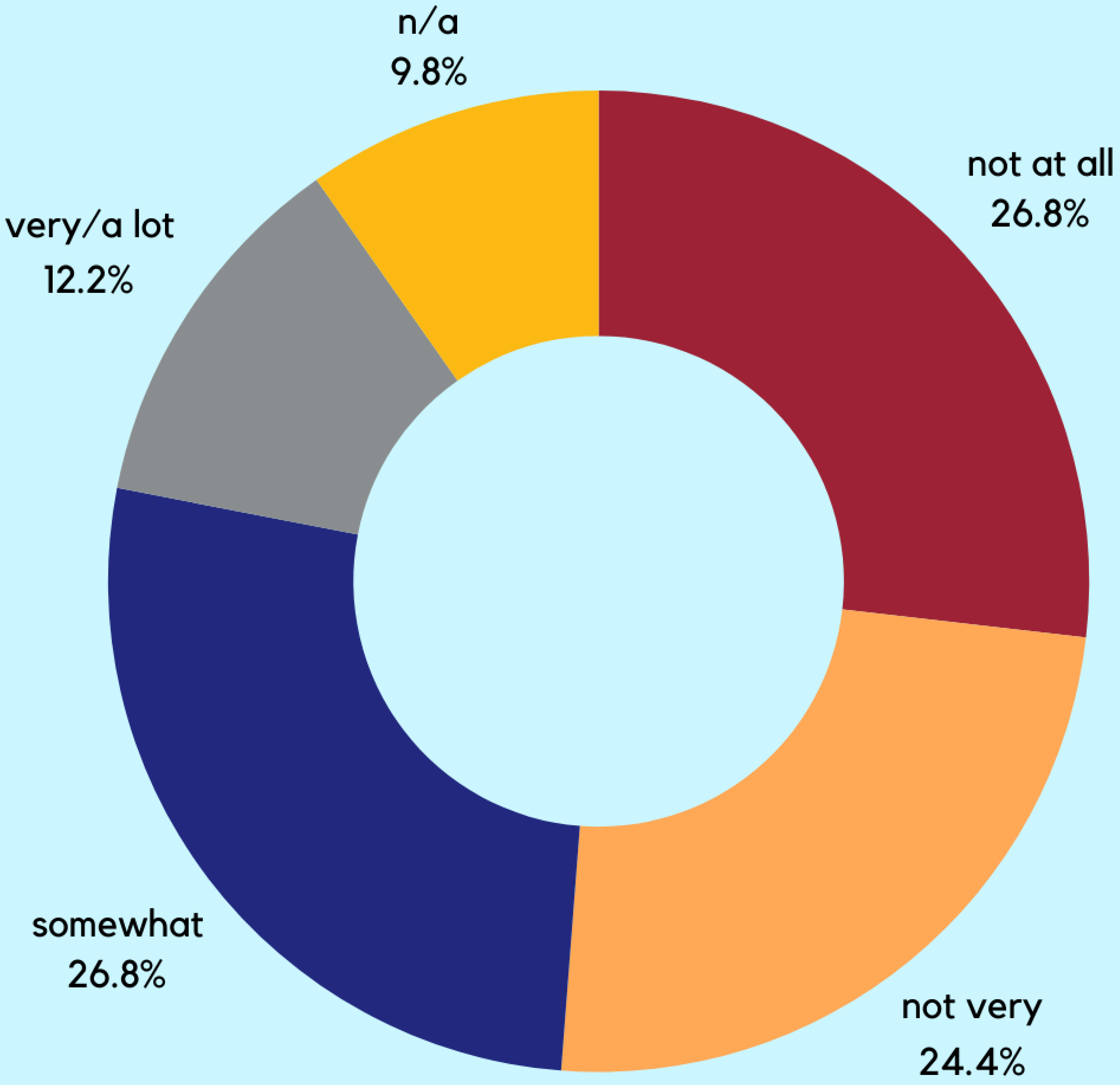

# Individuals: Students/Parents

“And my students are a very vocal, and I always try to teach them to really advocate for themselves, right that you want to be heard. And I think that's something that we're doing a good job, because they will let you know that they don't like something.” (HS Principal, FS)

“Or they'll bring their own or they have like tons of snacks, but they consider as a lunch, which is, that's what they see as lunch sometimes.” (HS Teacher/admin, FS)

“I don't if I don't eat the meal. I just drink the chocolate milk and if we have Apple or like fruit cup. I'll eat that too. So at least I got something in my stomach” (Student, Sat)

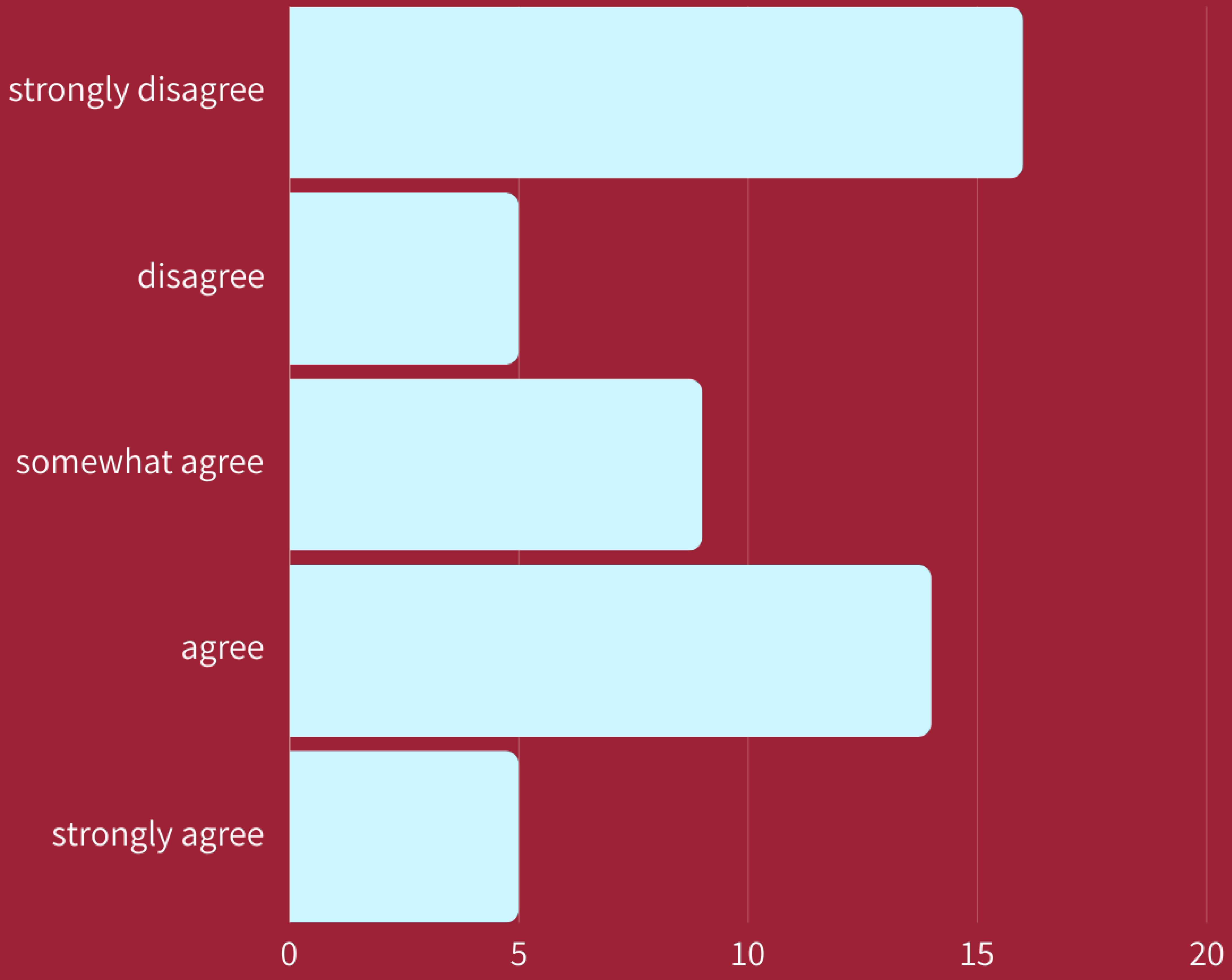


We asked **parents**: Does feeling stigmatized impact you/your child's decision to participate in school meals?

# Individuals: School Leadership

"And then when the kitchen staff was short, like when they had to move workers out, I was like, You know what, open the door, **let me go back and help them serve."**
(HS Principal, FS)

"Um yeah, with the principal and the climate staff. **They like to play a little music**, get the kids, you know, a happy lunch break."
(Teacher, FS)

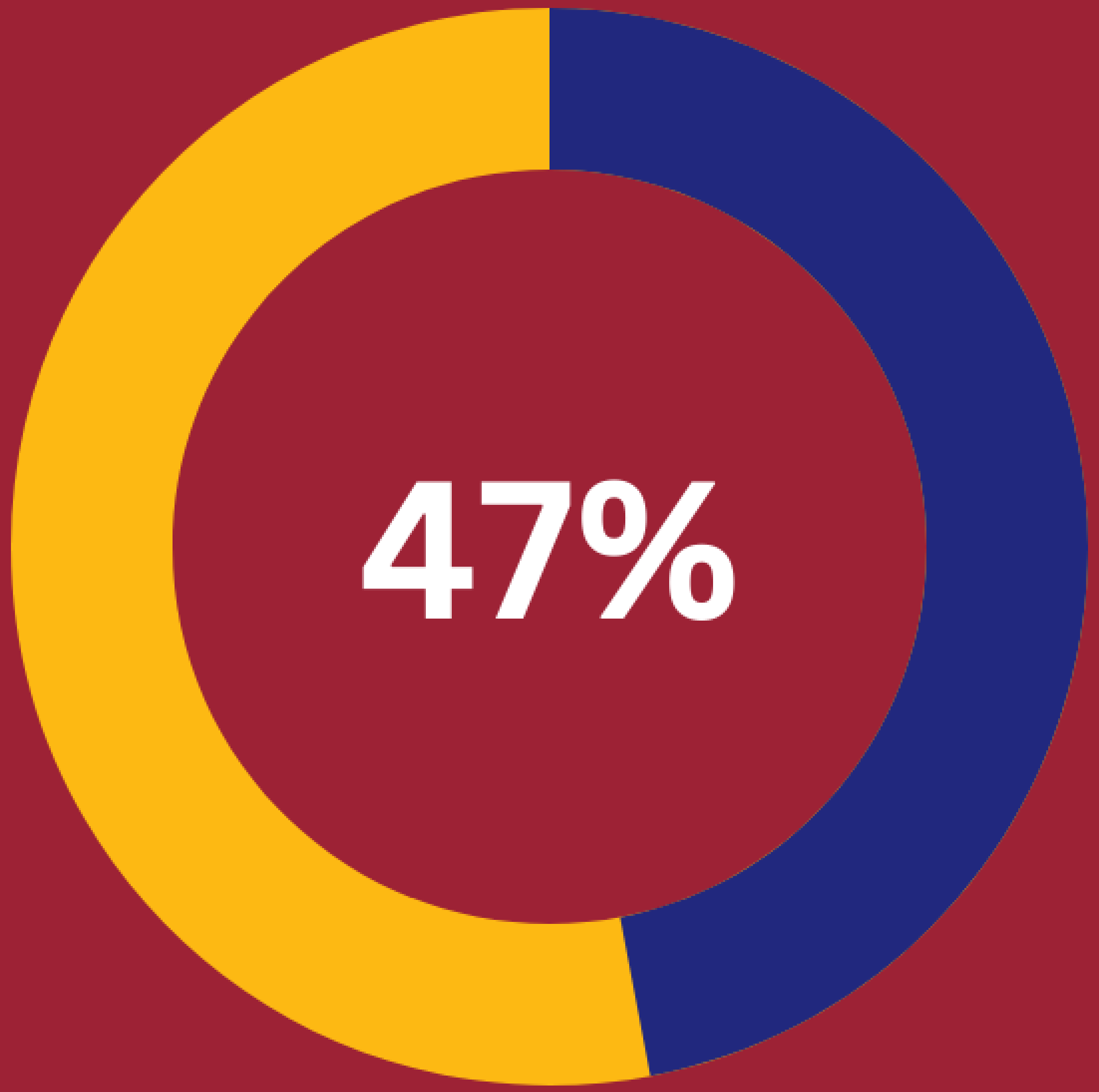


Of school teachers feel that their principal has taken an active role in the implementation of school meals.

# Inner Setting: Available Resources

"Yes. And how is the staffing right now? Are you at the staff you need? Or do you need?

I'm short 2. 3? I've been so long, I can't remember." (HS Food service, FS)

"So I feel like there needs to be a little extra. A little extra snack or something in their in their day. Besides just lunch and breakfast." (HS Food service, FS)

"Like we only got like five minutes at eat or 7 minutes.... it takes it takes four minutes to get everyone in line and get their lunch. Like it takes them to get to the lunchroom. And then they and then just like two minutes into the lunch room. Yes." (MS Student, FS)

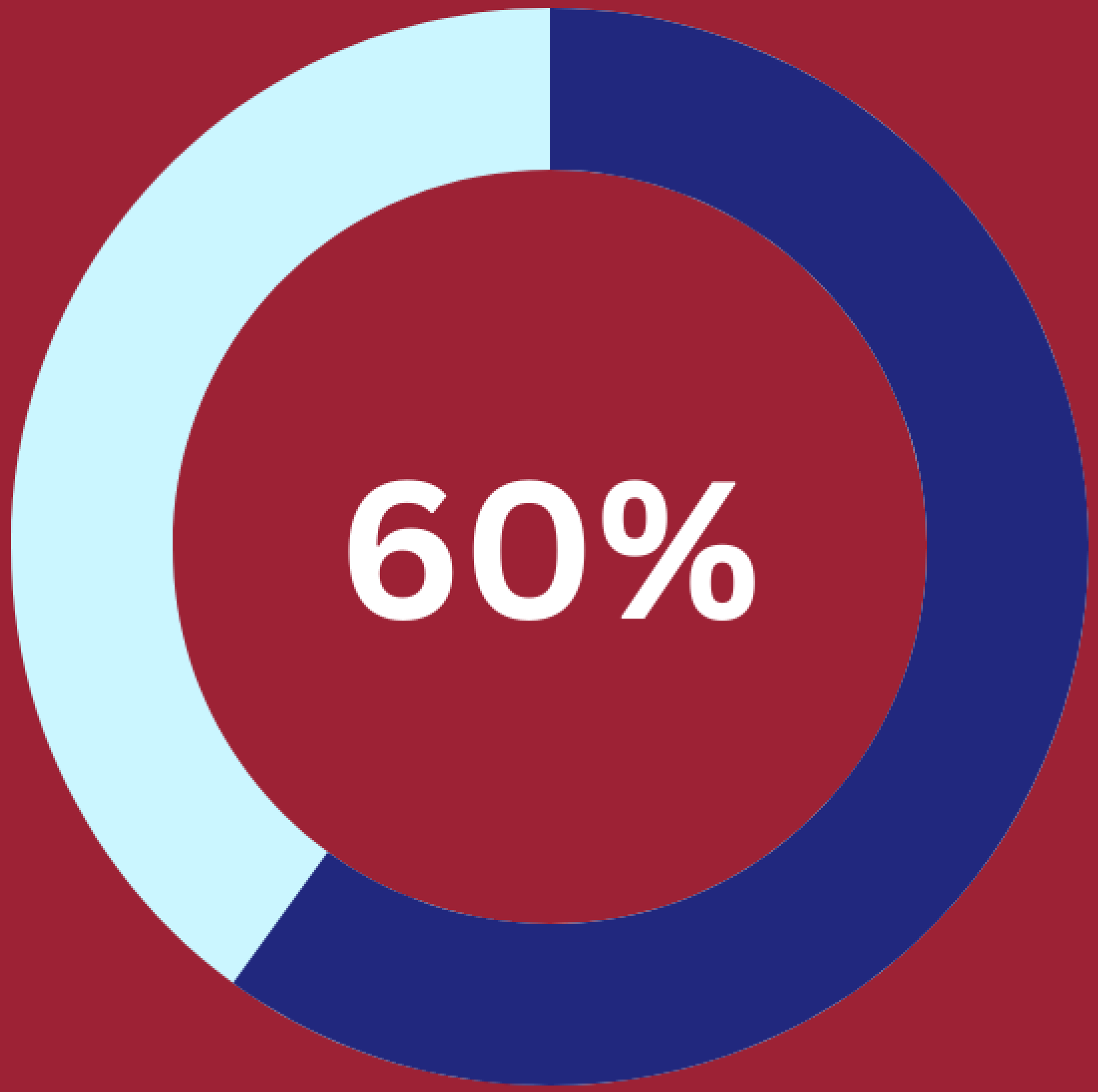


Of school teachers/staff feel that the school meals program requires more work than can be accomplished with current resources available to their school

# School Setting: Cafeteria Culture

“But for the kids like for them, it's social hour. They're always more focused on like, being with their friends. This is the first time throughout the day that they get to see all their friends from like, their different classes so it's always, it's always like a good vibe in there.”(Climate, Sat)

“They call it a freebie. Yeah. It's like, oh, you're getting you're getting the free lunch. You suck.” (HS Students, FS)

# District/Broader Setting: Local Attitudes

+

"In the last two years, it's become very inclusive and very like we're serving was what it is the thing falafels and stuff. Wow. Okay, the children so I know that the district as a whole it is reflecting a different cultures." (Parent, FS)

–

"Well, we have a lot of we have a lot of Spanish students here and they're not getting you know with what they will eat on a regular Yeah, they're they're actually getting...it's like American food. Yeah. Yeah, they're even more of American food opposed to you know, their culture." (Admin, FS)

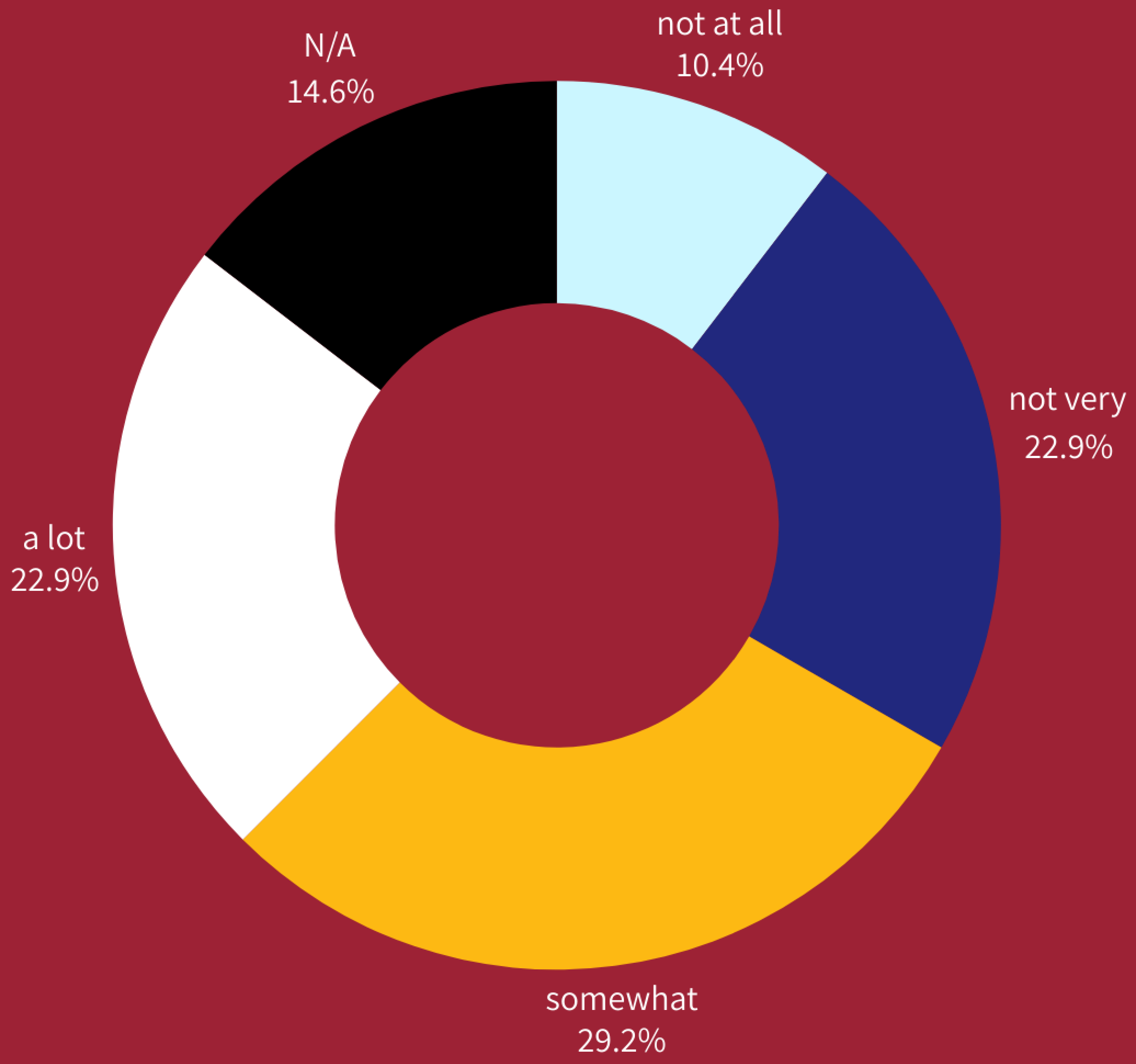


We asked parents if they feel that the school meals program takes the culture of the local area into account.

# Outer Setting: Local Environment

"Yeah, it's not many grocery stores around this area... it's a few corner stores that they received in fabulous bunch of junk, right? They don't so like fresh produce or things like that. It's always the quick meals, like the chicken fingers and fries, the sandwiches.  So I do not think it's a lot of grocery stores close in this area. So that can be an issue for some of our parents." (Teacher, FS)

"So if they were exposed to more food like that was a little bit Different. It's not really different, but they don't. They know junk food. They live around junk food, they eat junk food. They go to the corner store, they get their food. They know it. So they like it" (Teacher, sat)

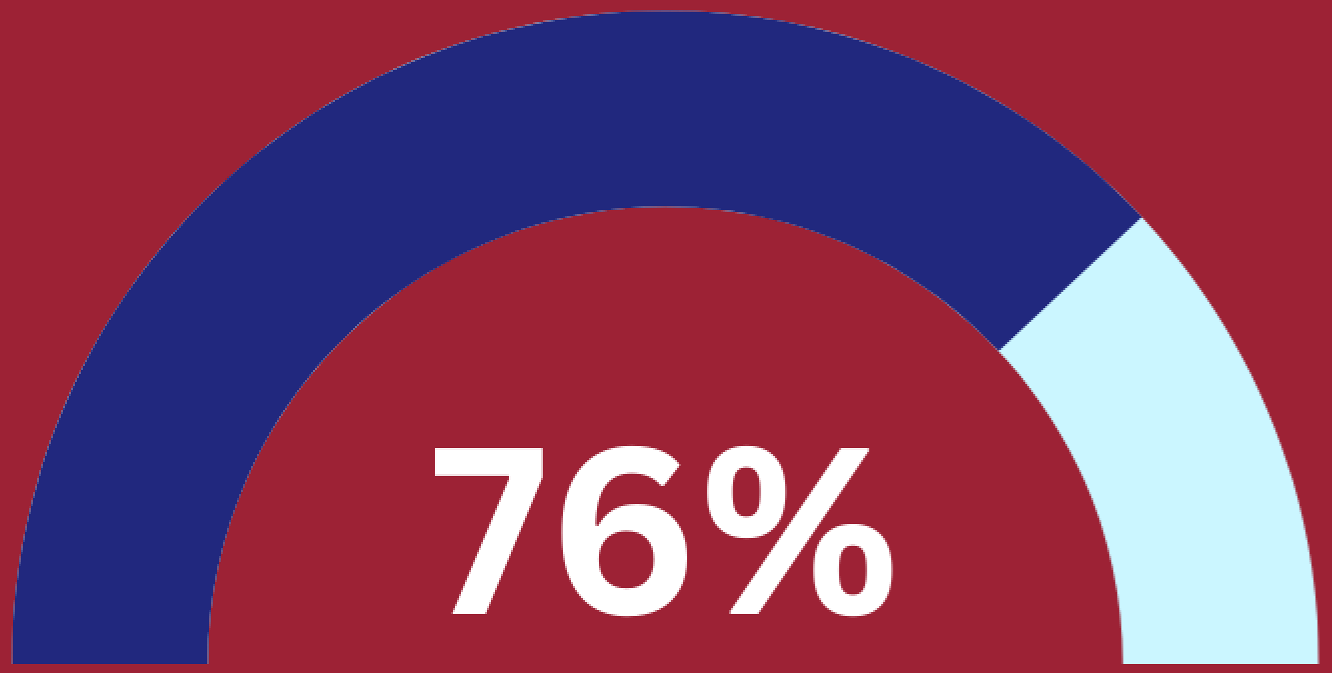


Of teachers either agree/strongly agree that that this policy improves the nutrition status of students who lack consistent access to food

# Barriers and Facilitators Across High- and Low-Participation Groups

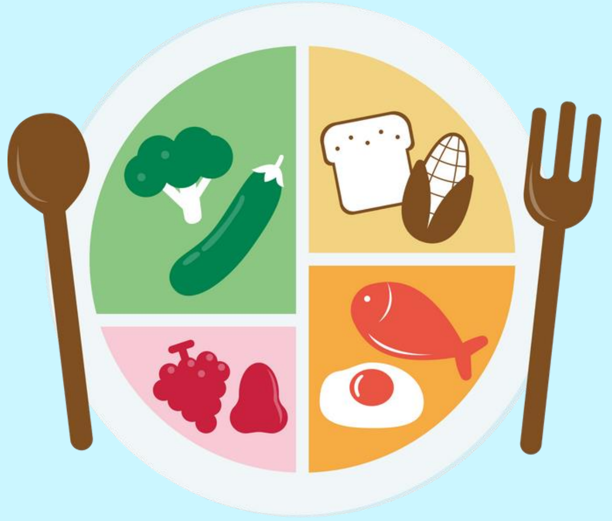

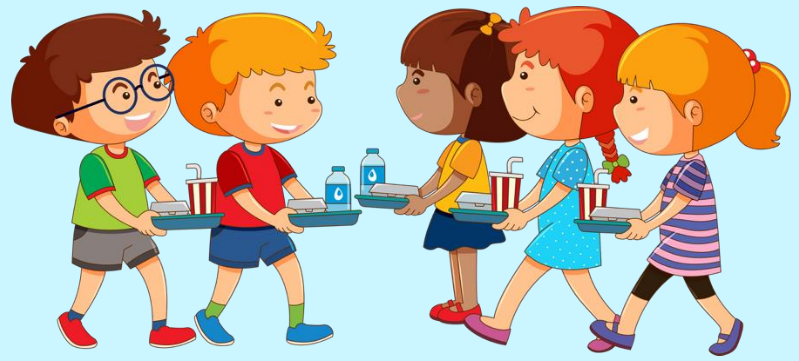

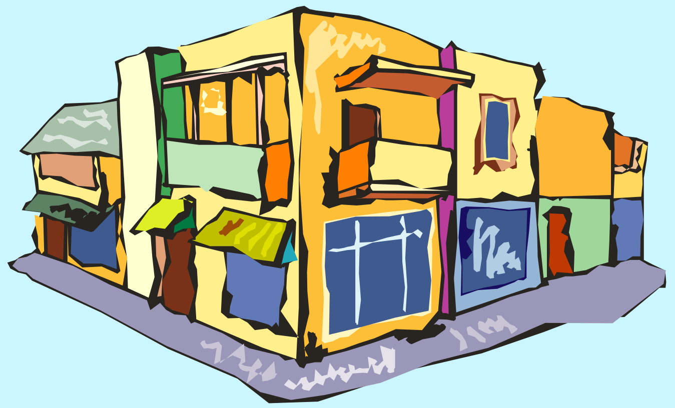

- Cafeteria Culture
    - Stigma, social culture
- Neighborhood Conditions
    - Corner stores, concerns with violence
- Need for Meals
    - Food insecurity and hunger, needs met

# Next Steps for the Study

## How we will use these findings?

- We are sharing the findings from this study across the city of Philadelphia, the United States, and internationally to raise awareness of school meals
- We are using these results to design and evaluate changes to implementation of school meals next year

## How can you get involved?

- We have a Community Advisory Board (CAB) and are building a community network listserv to share updates on the project. Want to get involved? Contact us below!

# Support from CAB and School District

While we have been working alongside the School District, we have built a Community Advisory Board of representatives of Community organizations, parents, and students that helped us to better understand the community we are working with

Laura Crandall
Dr. Jonathan Deustch
Irene Hong
Hillary Kane

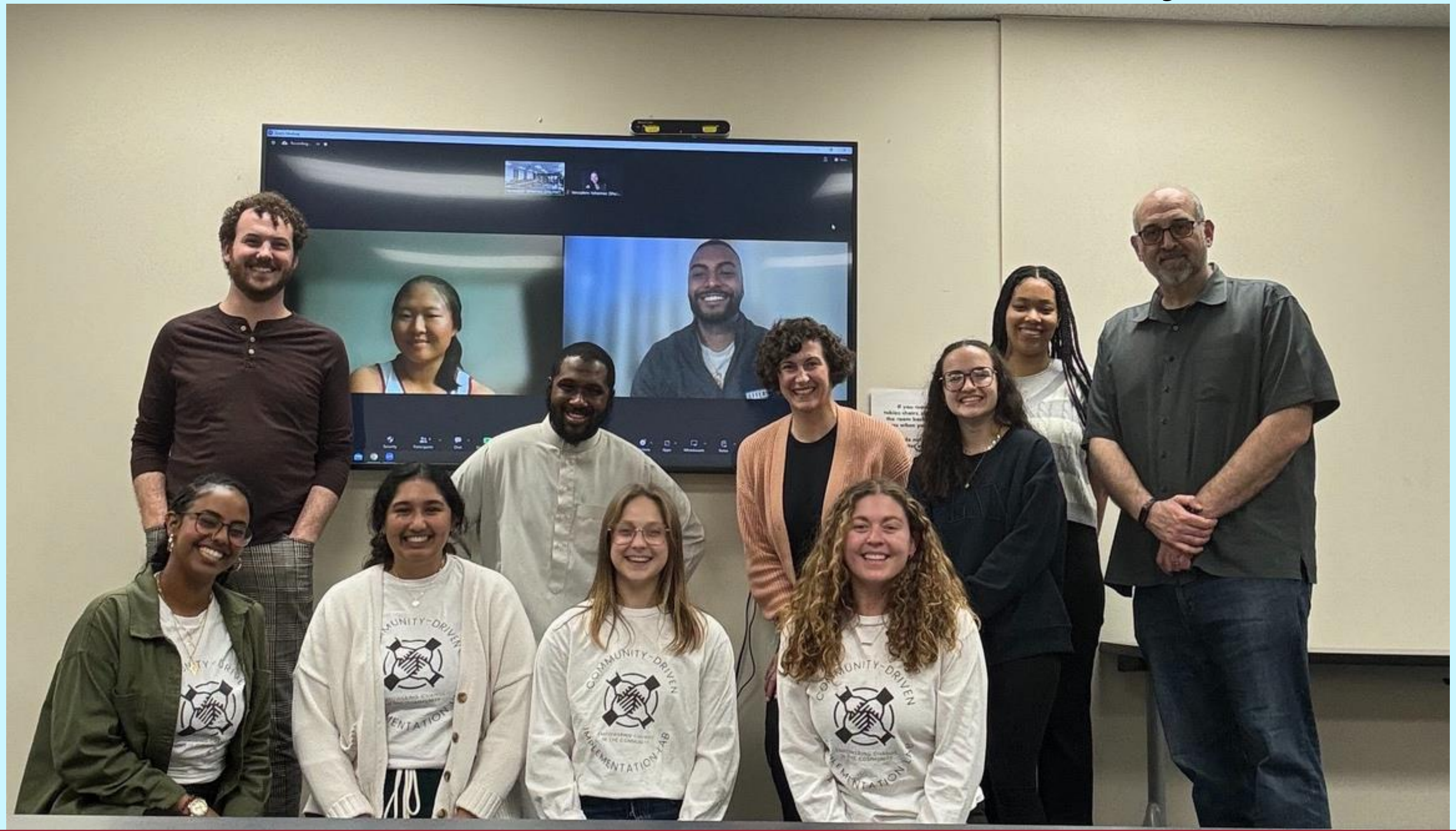

Jacob Kurtz
Rose Marone
Randy Persaud
Sultan Raheem

# School Meals at The School District of Philadelphia

**What do the students, parents, and staff at the school district think about school meals?**

## Who did we talk to?

We learned from 193 participants across 8 schools (6 elementary-middle, 2 high) in the district. Participants were varied in terms of race, ethnicity, and we had an equal distribution of roles.

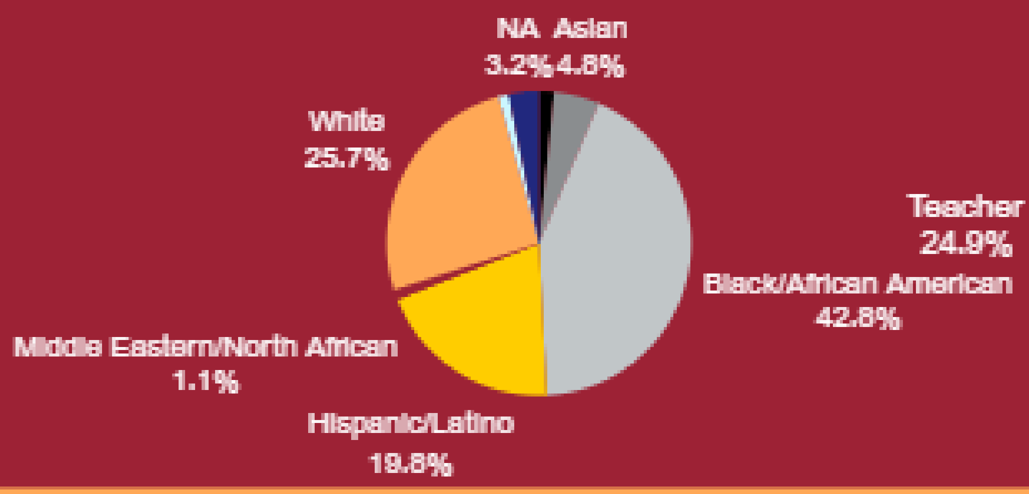


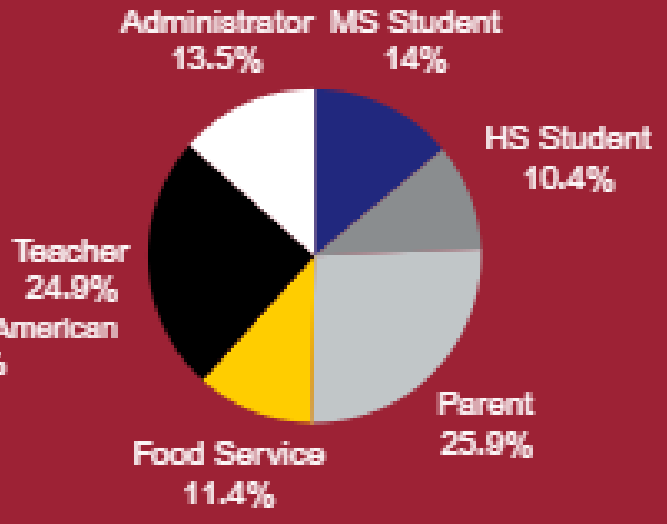


## What did we find?

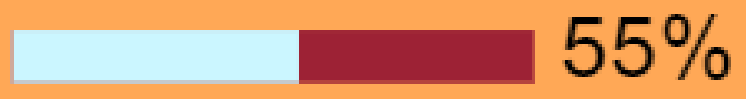


Of students feel that the school food is healthy (answered either "agree" or "strongly agree")

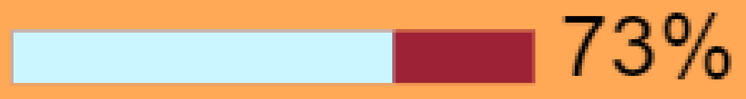


Of students feel school meals helps students struggling with hunger (answered either "agree" or "strongly agree")

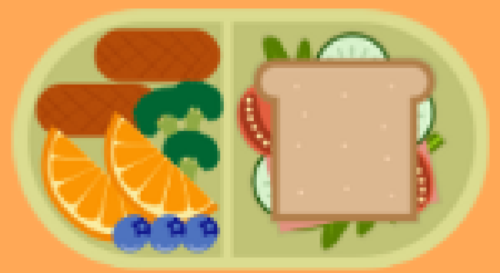

**'I feel like they put like other stuff on the plate that we don't eat instead of just giving us more of things we like.'
(MS Student)**

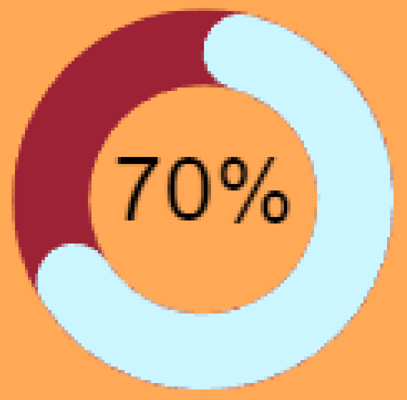


Students feel that they do **not** have a say in the foods included

# Most popular focus areas from the district

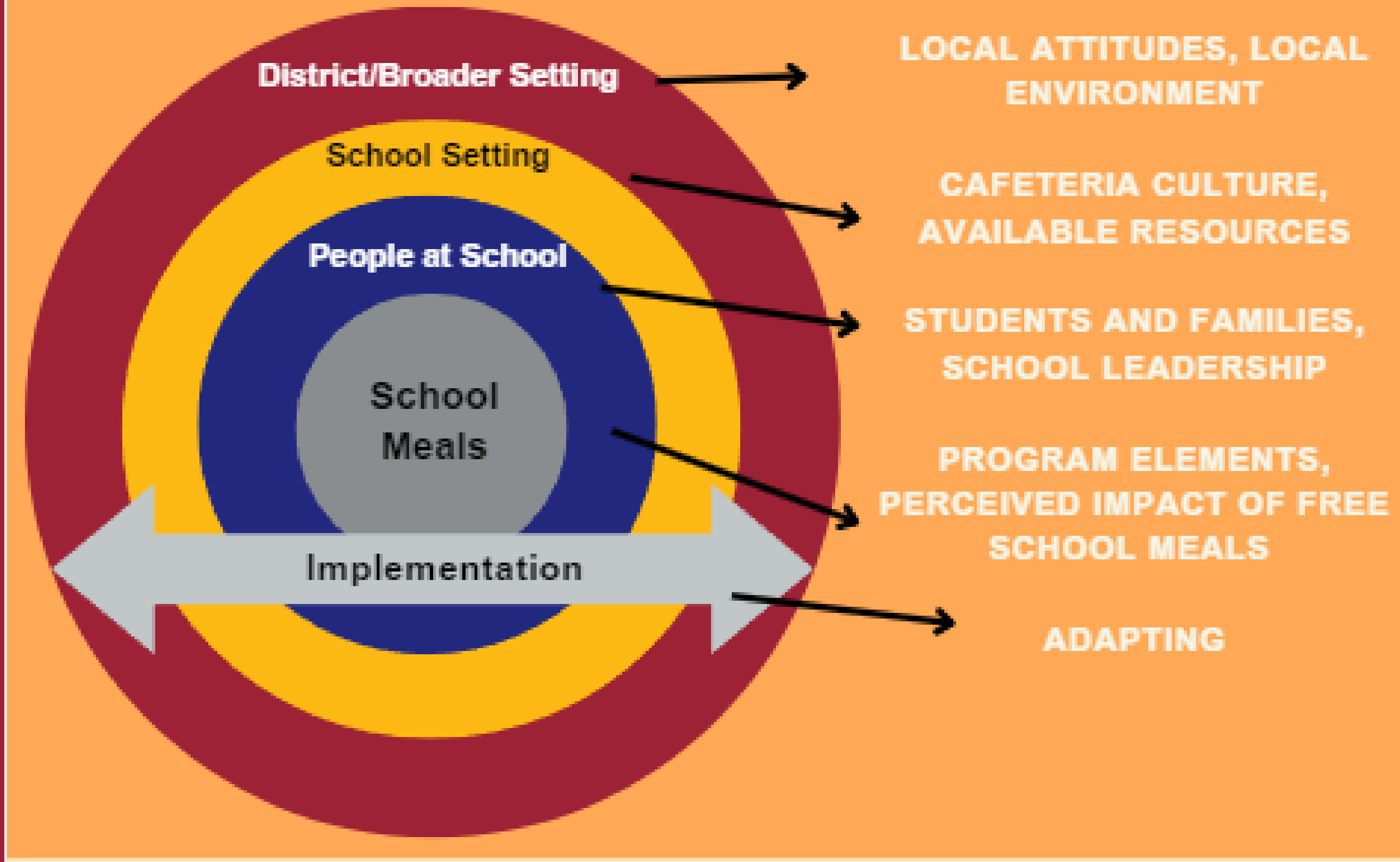


**Insights from Students**

'They call it a freebie. Yeah. It's like, oh, you're getting you're getting the free lunch. You suck.' - Cafeteria Culture

**Insights from Parents**

'In the last two years, it's become very inclusive and very like we're serving was what it is the thing falafels and stuff... so I know that the district as a whole it is reflecting a different cultures.' - Local Attitudes

**Insights from Staff**

'And I think that today, when they eat it, they're more focused. And clearly, they need it, right? Because they maybe haven't eaten yet. And it just gives them that little boost.' - Perceived Impact of Free Meals

# Thank you!

Questions!

implementationlab@temple.edu

gabriella.mcloughlin@temple.edu

# ACHIEVING EQUITABLE SCHOOL MEALS THROUGH A COMMUNITY-DRIVEN APPROACH

Divya Kulkarni, College of Science and Technology, Temple University
Molly Kerstetter MPH SBS, College of Public Health, Temple University
Yerusalem Yohannes MPH SBS, College of Public Health, Temple University
Garima Adhikari, College of Liberal Arts, Temple University
Gabriella M. McLoughlin PhD, College of Public Health, Temple University

Community-Driven Implementation Lab — Empowering change in the community
Temple University College of Public Health

***This project aims to identify what works well and what needs help in the implementation and participation of the universal school meals program in the School District of Philadelphia.***

## WHY DOES IT MATTER?

In 2020, almost 31 percent of households with children in Philadelphia County experienced food insecurity[1]. Addressing the implementation and quality of meals that students count on during school is essential to maximizing consumption of nutritious meals.

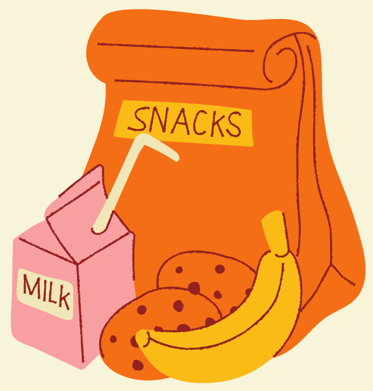


## WHO ARE WE AND WHAT ARE WE DOING?

- Dr. McLoughlin's Community-Driven Implementation Science Research Lab conducted an NIH funded study to address gaps in implementation of the Philadelphia school meals program.
- In September of 2023, our team recruited eight schools across the Philadelphia School District. They conducted surveys, interviews, and focus groups with students grades 6-12, as well as parents, teachers, climate staff, administration, and food service staff. Essential demographics and findings of this study are highlighted below.

### INTERVIEW PARTICIPATION

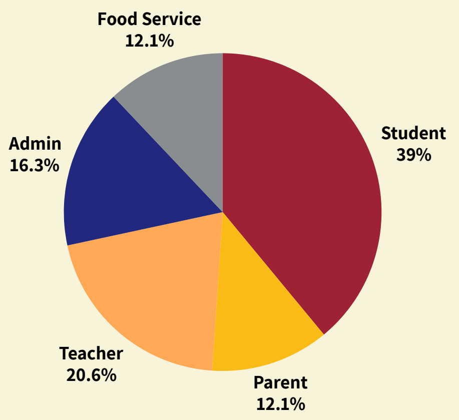


### SURVEY PARTICIPATION

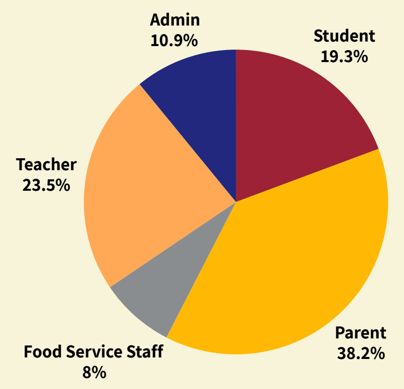


### TOTAL PARTICIPATION

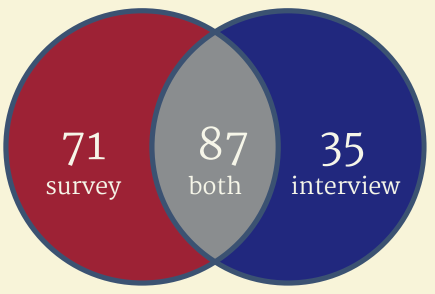


We observed over 30 meals, received 158 surveys, and conducted 122 interviews.

### SURVEY SAMPLE OF STUDENTS: GRADE LEVELS

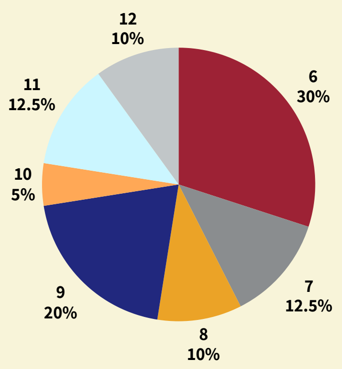


### RACIAL/ETHNIC BREAKDOWN OF ENTIRE STUDY SAMPLE

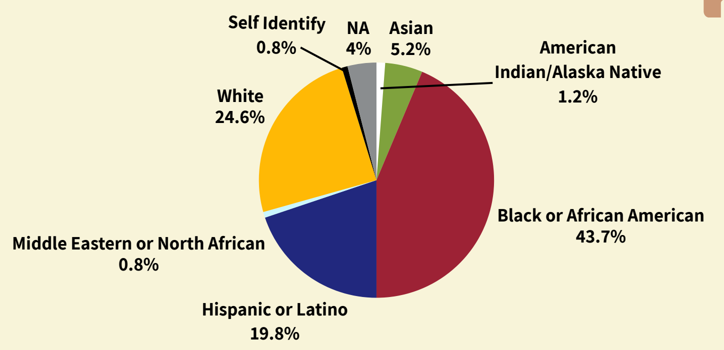


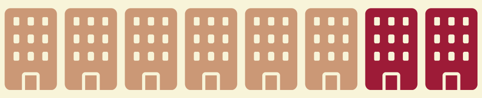

Eight public schools

- Six middle schools
- Two high schools
- Six full-service kitchens
- Two satellite kitchens

## IMPACTFUL QUOTES FROM PARTICPANTS

"The benefits of free lunch and free breakfast is it comes in handy because **a lot of parents right now are, you know, living under their needs, you know, living paycheck to paycheck**. Sometimes parents don't got, you know, food in their fridge to feed their kids. So **being able to go to school and get two free meals is important**." (Parent)

"They call it a **freebie**. Yeah. It's like, oh, you're getting **you're getting the free lunch. You suck**." (HS Students)

"We can tell after a weekend who's hungry who didn't eat at home. **You can tell who doesn't eat dinner** and comes in and devours breakfast." (Food Service)

"And my students are a very vocal, and **I always try to teach them to really advocate for themselves**, right that you want to be heard. And I think that's something that we're doing a good job, because **they will let you know that they don't like something**." (Admin)

## IMPORTANT FINDINGS IN THE DATA

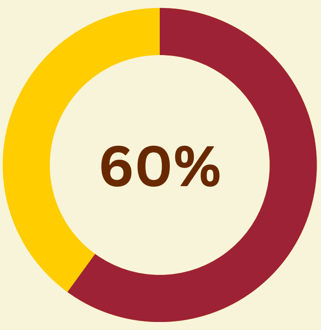


of school staff, including teachers, admin, and food service, feel that the school meals policy requires more work than can be accomplished with current resources available to their school

Levels of agreement when students and parents are asked if the school meals program helps the low-income students in school access healthy food:

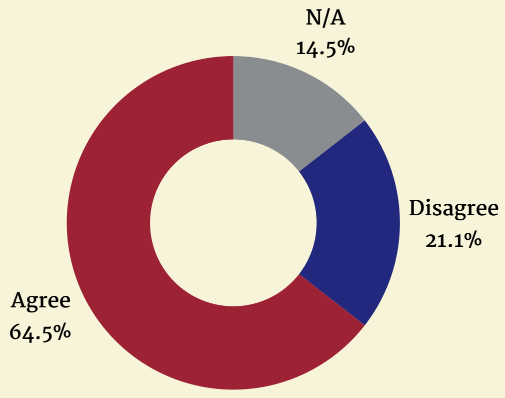


Almost 65 % of all participants agree that the school meals program helps the most socioeconomically disadvantaged students in your local school access nutrition support.

1. Philabundance. (2022, July 20). Food Insecurity Rates for Our Service Area. Philabundance. Retrieved May 20, 2024, from https://www.philabundance.org/food-insecurity-rates-for-our-service-area/

# MAIN TAKEAWAYS

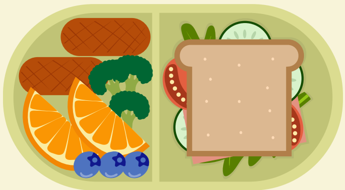

- Major barriers include the quality and cultural appropriateness of meals, a lack of resources, and stigma surrounding free meals in older grades:

Levels of agreement when students are asked if the school meals program makes students from different cultures feel included:

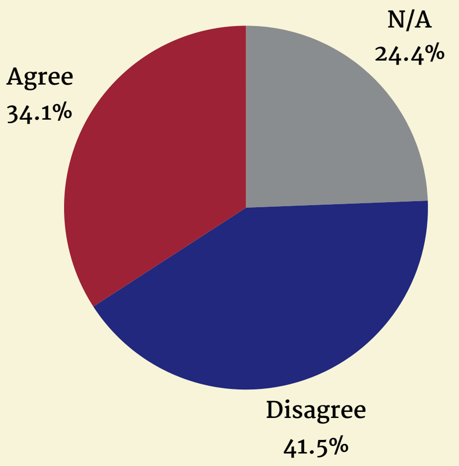


Levels of agreement when all participants are asked if the school meals program reduces stigma associated with receiving free school meals:

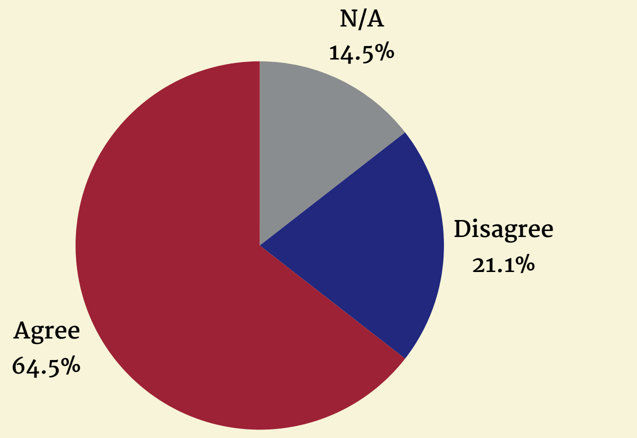


Questions were asked on a scale with choices ranging from strongly disagree, disagree, agree, strongly agree, and N/A. Levels of agreement were grouped for analysis purposes.

- Main facilitators are perceptions of school administrators and a definite need for school meals across all participant groups and schools:

Levels of agreement when all participants are asked if the school meals program helps students who are struggling with hunger:

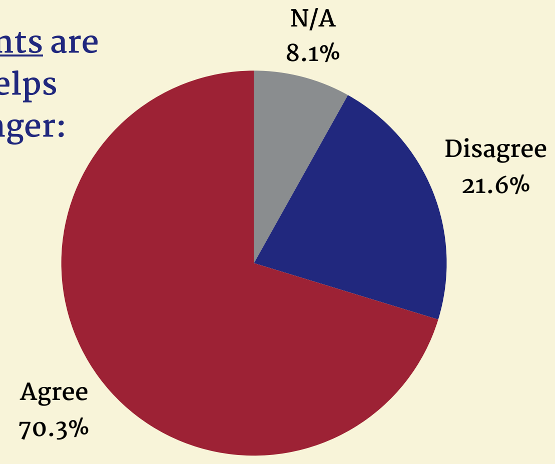


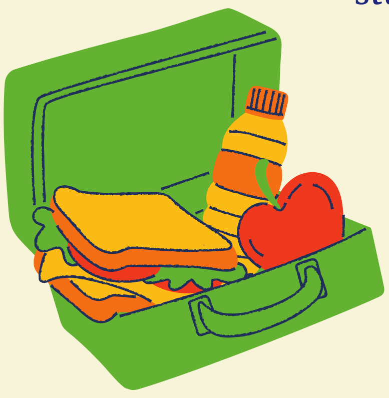

# CONCLUSIONS

A community-based approach to school meals allowed this research team to identify the tiers of decision making and influence that ultimately affect delivery of school meals. Engaging with students, parents, teachers, admin, and food staff helped us create a holistic picture of the school meal system:

- Factors such as access to meals, genuine implementation leaders like food staff and admin, relative culture and attitude of school towards meals are all working well.
- Factors such as quality and recipient-centeredness of meals need some work.

# WHAT'S NEXT?

- We are sharing the findings from this study across the city of Philadelphia, the United States, and internationally to raise awareness of school meals.
- We are using these results to design and evaluate changes to implementation of Universal School Meals and meal policy.
- The next part of this project will explore barriers and facilitators of school meals at the district level.

# ABOUT US!

We are a research Team at Temple called the Community-Driven Implementation Science lab.
What does this mean?
We do research that is driven by the needs of the community by partnering with local experts and people who are often forgotten in research.
We use a range of methods aimed at improving the delivery of evidence-based programming!

About our Community Advisory Board
We led this project in partnership with our Community Advisory Board (CAB).
Here are our members and their roles:

Laura Crandall, Office of Children and Families
Dr. Jonathan Deustch, Drexel Food Lab
Irene Hong, Feeding Philly/Student
Hillary Kane, PHENND/Parent
Jacob Kurtz, Reinvestment Fund
Rose Marone, Feeding Philly/Student
Randy Persaud , Jefferson
Sultan Raheem, Parent Representative

COMMUNITY-DRIVEN IMPLEMENTATION LAB
EMPOWERING CHANGE IN THE COMMUNITY

# WANT TO GET INVOLVED?

We have a Community Advisory Board (CAB) and are building a community network listserv to share updates on the project. Want to get involved? Contact us here:

Contact us!
